# Functionalized Reduced Graphene Oxide‐Based Nanocomposite Hydrogels for Enhanced Osteogenesis in Bone Tissue Engineering

**DOI:** 10.1002/adhm.202501941

**Published:** 2025-08-07

**Authors:** George Mihail Vlăsceanu, Cătălina Ionela Zamfir, Cornel Baltă, Hildegard Herman, Alina Ciceu, Maria Alexandra Duma, Mariana Ioniță, Roxana‐Cristina Popescu, Anca Hermenean

**Affiliations:** ^1^ Faculty of Medical Engineering National University of Science and Technology Politehnica Bucharest Gheorghe Polizu 1–7 Bucharest 011061 Romania; ^2^ Advanced Polymer Materials Group National University of Science and Technology Politehnica Bucharest Gheorghe Polizu 1–7 Bucharest 011061 Romania; ^3^ Aurel Ardelean Institute of Life Sciences Vasile Goldis Western University of Arad 94–96 Revolutiei Av. Arad 310025 Romania; ^4^ eBio‐Hub Research Centre National University of Science and Technology Politehnica Bucharest‐Campus Iuliu Maniu 6 Bucharest 061344 Romania; ^5^ National Institute for Research and Development in Physics and Nuclear Engineering “Horia Hulubei” Department of Life and Environmental Physics 30 Reactor Magurele 007125 Romania; ^6^ Faculty of Medicine Vasile Goldis Western University of Arad 94–96 Revolutiei Av. Arad 310025 Romania

**Keywords:** bone regeneration scaffolds, ectopic osteogenesis, functionalized reduced graphene oxide, microCT‐based mineral analysis, nanocomposite hydrogel biomaterials, subcutaneous pocket implantation

## Abstract

This study presents the development of nanocomposite hydrogels that integrate functionalized reduced graphene oxide (rGO) derivatives carboxylated (CBX) and aminated (AMN) in combination with gellan gum, gelatin, and cellulose nanofibrils (CNFs) to enhance osteogenesis for bone tissue engineering. Their synthesis involves ultrasound exfoliation, genipin crosslinking, and freeze‐drying. Micro‐computed tomography (µCT) reveals highly porous, interconnected architectures facilitating nutrient diffusion and cell infiltration. CBX/AMN scaffolds exhibit superior hydration capacity and a biphasic degradation profile. Spectroscopically, enhanced interfacial interactions were confirmed, contributing to the overall structural stability of the hydrogels. In vitro, all scaffolds are cytocompatible, sustain cell viability, and promote osteogenic differentiation consistent with adaptive cellmaterial interactions. Subcutaneous implantation in mice confirms scaffold biocompatibility, showing no signs of inflammation or foreign body response. Histological and immunofluorescence analyses reveal osteogenic potential, with enhanced mineralization and elevated expression of osteopontin and osteocalcin. µCT imaging of explants quantifies ectopic mineralization, spatial deposition patterns, and bone mineral density, correlating scaffold architecture and composition with osteogenic performance. These findings suggest that the synergistic integration of functionalized rGO, CNFs, and biopolymers results in scaffolds with enhanced structural and biological properties, highlighting their potential for design optimization and clinical translation in bone regenerative medicine.

## Graphical abstract

Charge‐opposed reduced graphene oxide fillers are co‐integrated into biopolymeric nanocomposite scaffolds, synergistically enhance osteogenesis. Multiscale characterization reveals how surface chemistry and porosity dictate ectopic mineral architecture. In vivo micro‐computer tomography and histology confirm improved biomineralization and bone mineral density, positioning dual‐filler systems as superior alternatives to single‐charge strategies for regenerative bone engineering.

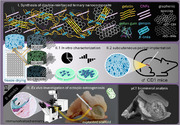

## 1 Introduction

The development of advanced nanocomposites has attracted significant attention in recent years due to their transformative potential in regenerative medicine and tissue engineering. Among these, nanocomposite hydrogels have emerged as promising candidates for bone tissue engineering applications due to their biocompatibility, mechanical strength, and ability to mimic the extracellular matrix (ECM).^[^ ^1^ ^]^ Despite notable advances, current hydrogels often fail to achieve an optimal balance between mechanical strength and bioactivity, limiting their practical translation to broader applications. Integrating natural polymers with nanoscale reinforcements provides an opportunity to engineer materials with enhanced structural integrity, controlled degradation, and favorable cellular interactions.^[^ ^2^ ^]^ To address the critical challenges of bone regeneration, we hypothesize that the inclusion of functionalized reduced graphene oxide (rGO) derivatives will enhance the mechanical integrity, controlled biodegradability, and osteogenic potential of gelatin‐based hydrogels, thereby providing a versatile scaffold for bone regeneration.

Graphene and its derivatives, particularly rGO, have attracted considerable interest for their unique physicochemical properties, including high mechanical strength, electrical conductivity, and large surface area.^[^ ^3^, ^4^, ^5^ ^]^ Functionalization of rGO with chemical groups such as carboxyl ^[^ ^6^ ^]^ and amino groups ^[^ ^7^ ^]^ further enhances its compatibility with polymeric matrices and broadens its application spectrum. Incorporating these functionalized graphene derivatives into hydrogel matrices has been shown to modulate key properties such as porosity, swelling behavior, and degradation rate, thereby making them highly suitable for bone regeneration applications.^[^ ^5^, ^8^, ^9^ ^]^

Natural polymers, such as gelatin and gellan gum, are widely used in hydrogel formulations due to their biocompatibility, biodegradability, and ability to support cell adhesion and proliferation.^[^ ^10^, ^11^, ^12^, ^13^ ^]^ Gelatin, derived from collagen, mimics the native ECM, providing biochemical cues essential for tissue repair.^[^ ^11^, ^14^ ^]^ Gellan gum, a microbial polysaccharide, contributes mechanical stability and forms thermoreversible hydrogels, making it an excellent complementary component,^[^ ^15^, ^16^, ^17^ ^]^ especially since it shares similarities with glycosaminoglycans^[^ ^18^ ^]^ in the bone extracellular matrix, while the calcium ions used to crosslink it play a critical role in osteogenic signaling.^[^ ^19^ ^]^ Furthermore, cellulose nanofibers (CNFs), obtained through chemical modification of natural cellulose, add another dimension of reinforcement due to their high tensile strength, hydrophilicity, strong intermolecular hydrogen bonds, and biocompatibility.^[^ ^20^, ^21^, ^22^ ^]^ Their fibrous structure forms a robust and interconnected network, closely mimicking the architecture of collagen fibers found in the native extracellular matrix.^[^ ^23^ ^]^ Despite being rather biologically inert and lacking intrinsic osteogenic cues, these materials effectively support tissue integration without eliciting significant inflammatory responses.^[^ ^24^ ^]^ Moreover, they have the unique ability to accommodate intermediate tissue stages during the degradation process, thereby providing a temporal scaffold that facilitates progressive tissue regeneration during their prolonged functional lifespan in physiological environments, since compared to natural polymers, they exhibit superior resilience to enzymatic and hydrolytic degradation.^[^ ^25^ ^]^ Synergistically, CNFs create a flexible yet strong matrix,^[^ ^26^ ^]^ while rGO introduces rigidity and reinforcement at the microlevel.^[^ ^27^, ^28^, ^29^ ^]^ This balanced mechanical profile supports cell attachment, proliferation, and differentiation under physiological loading conditions.^[^ ^30^ ^]^ This dual effect enables the nanocomposite to deliver biochemical cues in a controlled manner while ensuring scaffold stability during the initial healing phase but degrades at a rate harmonized to tissue remodeling.^[^ ^31^ ^]^

This study presents a novel approach to designing hydrogels for bone tissue engineering by incorporating functionalized rGO derivatives into a composite matrix comprising gelatin, gellan gum, and CNFs. These multi‐component hydrogels were systematically investigated to understand the synergistic effects of the constituents on the material's structural, swelling, and degradation properties. The incorporation of rGO functionalized with carboxyl (CBX) and amino (AMN) groups introduces distinct interfacial interactions that influence the mechanical and biological performance of the hydrogels.^[^ ^32^, ^33^, ^34^ ^]^ While graphene oxide has shown promise in promoting osteogenesis,^[^ ^35^, ^36^ ^]^ less is known about rGO and specific functional groups efficacy in complex tissue environments. To the best of our knowledge, this is the first study to integrate surface‐charge‐distinct carboxylated (CBX) and aminated (AMN) rGO derivatives with gelatin, gellan, and CNFs to design a synergistic platform that addresses both mechanical and biofunctional demands in bone regeneration.

The aim of this study is to develop and evaluate nanocomposite hydrogels incorporating functionalized reduced graphene oxide derivatives, gellan, gelatin, and cellulose nanofibrils to enhance osteogenesis for bone tissue engineering. The study seeks to address both mechanical and biofunctional challenges in bone regeneration by engineering scaffolds with improved structural integrity, controlled degradation, and osteogenic potential. It explores the synergy between the components and their ability to promote cell attachment, proliferation, differentiation, and mineralization, providing a foundation for future clinical applications in bone repair and regenerative medicine. To assess the intrinsic osteoinductive properties of the developed scaffolds, an in vivo subcutaneous implantation model was employed, enabling evaluation of mineralization and osteogenic marker expression in an ectopic environment.

## 2 Results

### 2.1 Morpho‐Structural Properties

#### 2.1.1 Micro‐Computer Tomography (µCT)

**Figure 1:**
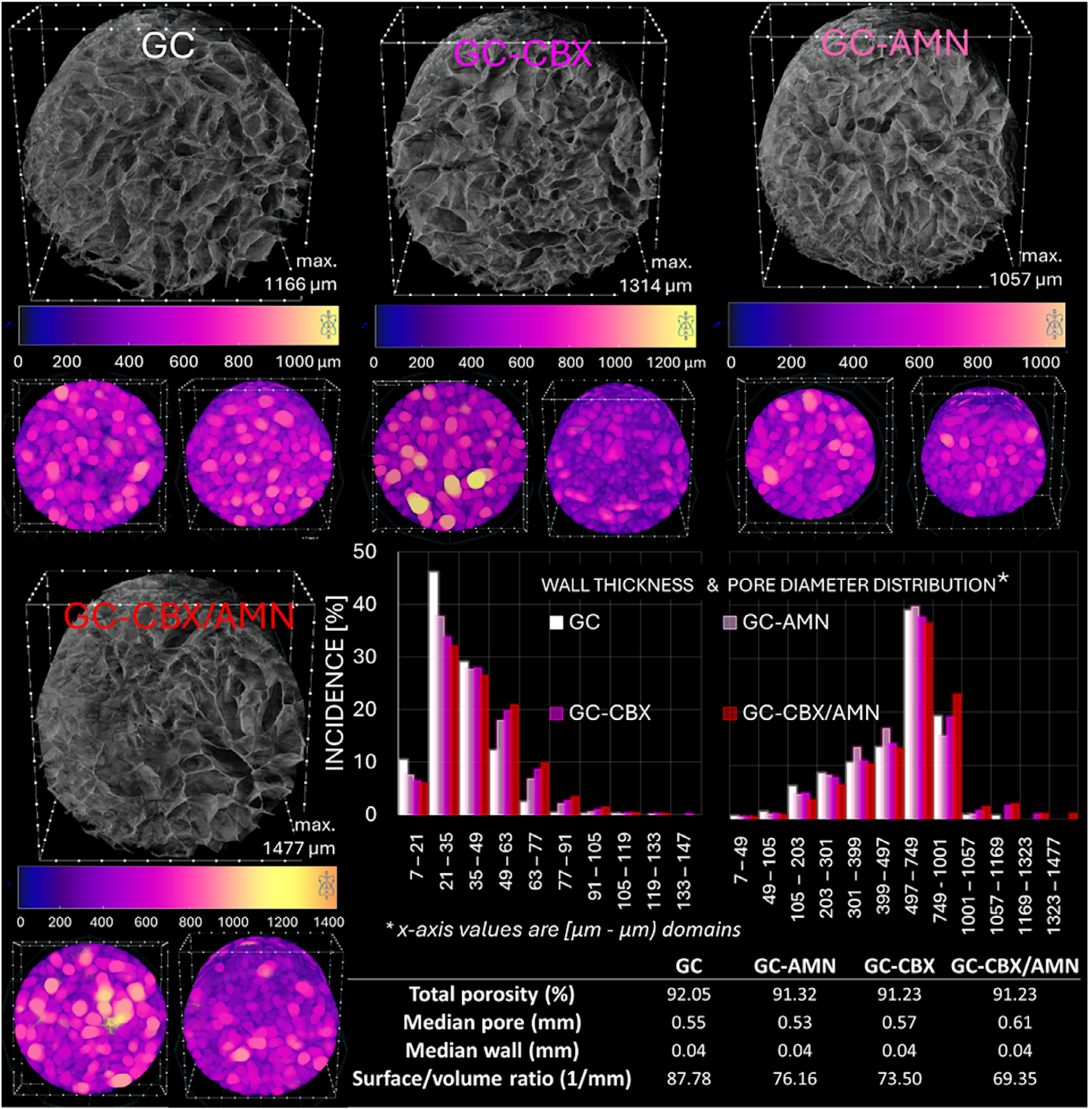
Morphological characterization of freeze‐dried nanocomposites using X‐ray microtomography. 3D tomographic reconstructions of GC, GC‐AMN, GC‐CBX, and GC‐CBX/AMN, as well as color‐coded pore size distributions obtained from µCT analysis and visualized as 3D objects. Plotted size domains for pore diameters and wall thickness. Tabulated quantitative analysis of the nanocomposite microstructure.

Following freeze‐drying, µCT was employed to evaluate the morphological characteristics of the four nanocomposites from both qualitative and quantitative perspectives. The results, along with visual representations and a summary table of supporting data, are presented in **Figure** **1**. The black‐and‐white tomograms reveal the highly porous and interconnected nature of the four samples, showcasing pores of varying sizes, with an average porosity of 91.4%. Qualitative analysis focused on assessing pore diameters and the distribution of solid sample thicknesses, as depicted in the lower right corner of the figure. The numerical analysis was conducted relative to the scanning resolution of the samples (7 µm), with data reported across multiple values of this constant on the *x*‐axis. The results are grouped into various intervals to enhance clarity and facilitate interpretation.

In addition to the numerical data, color‐coded tomograms were generated and presented alongside a color scale bar, offering an intuitive depiction of the pore distribution within the specimens. This visualization enhances the understanding of pore architecture, enabling a more detailed analysis of how pore size and distribution vary among the different nanocomposites. The color scale effectively highlights variations in pore dimensions, facilitating interpretation of the structural characteristics of the samples. The color‐coded representation associated with the tomograms obtained through phase inversion shows the range of values for pore sizes with various upper‐limits: 1166 µm for the GC, 1057 µm for GC‐AMN, 1314 µm for GC‐CBX, and 1477 µm for the GC‐CBX/AMN.

The GC sample exhibits wall thicknesses predominantly within the range of 21–35 µm, with no significant variations toward greater thickness. In contrast, matrices containing functionalized reduced graphene oxide, GC‐AMN and GC‐CBX demonstrate a clear trend toward the formation of thicker walls, indicating the influence of these compounds on wall structure, although the average is consistent throughout the batch at ≈40 µm. The GC‐CBX/AMN matrix demonstrates the broadest distribution for wall thickness, suggesting a synergistic effect from the combined presence of amino and carboxyl functional groups. This is likely attributed to enhanced electrostatic interactions between the nanocomposite components during the pre‐drying stage of fabrication, promoting the formation of thicker walls.

According to the pore size distribution graph, the presence of functionalized reduced graphene oxide in the matrix structures significantly influences pore structure compared to the control matrix. In the control sample, pores are predominantly concentrated within the range of 497–749 µm. The rGO‐AMN and rGO‐CBX reinforcements result in a more varied distribution of pore sizes, with significant presence in smaller ranges, indicating an expansion in the distribution of pore sizes. The GC‐CBX/AMN formulation exhibits broadened pore distribution, including larger pore sizes, suggesting a cumulative effect of these two types of carbonaceous materials filling in, promoting the formation of larger pores.

#### 2.1.2 Fourier Transform Infrared (FTIR)

**Figure 2:**
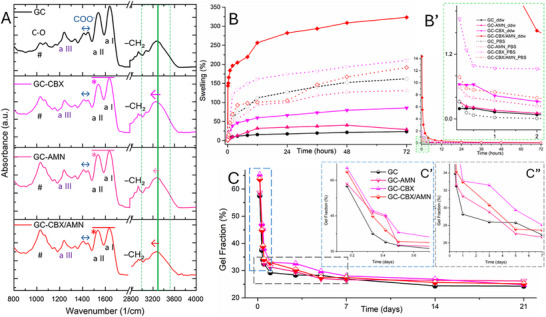
Characterization of the nanocomposites. A) Fourier Transform Infrared (FTIR) spectra of the GC, GC‐AMN, GC‐CBX, and GC‐CBX/AMN formulations. B) Swelling behavior of the hydrogels in deionized water (ddw) and phosphate‐buffered saline (PBS). Inset B' shows the swelling kinetics over time. C) Gel fraction of the hydrogels after enzymatic degradation. Insets C' and C" highlight specific timeframes of the degradation process.

FTIR analysis was employed to evaluate the chemical composition of the nanocomposite and to probe potential interfacial interactions between the matrix and nanofiller phases. Considering the subtle differences between the formulations, only minor variations were expected to appear (**Figure** **2**A).

The O─H stretching vibrations (3500–3000 cm^−1^) emerge as some of the most prominent signals due to the abundance of hydroxyl groups in gelatin, gellan gum, and CNF. rGO‐AMN and rGO‐CBX interact with hydroxyl groups, forming hydrogen bonds that toughen the nanocomposite network, lower the vibrational frequency of the O─H, and redshift (Figure 2A, ←) the maximum (|) absorbances in the region while also broadening the signal altogether.^[^ ^37^ ^]^ The concurrent employment of rGO‐AMN and rGO‐CBX further reduced O─H absorption, indicating enhanced interactions between functional groups from the rGO and hydroxyl functionalities.

Moving on to lower wavenumbers, C─H stretching vibrations (3000–2800 cm^−1^) depict a band associated with the stretching of methyl (─CH_3_) and methylene (─CH_2_) groups in all samples with slightly varying intensities. Minor intensity differences of the peaks are attributed to variations in rGO functionalization, particularly for the case of GC‐AMN which feature the highest amount of aliphatic chains in its structure.^[^ ^38^ ^]^

The amide I (a I) 1650 cm^−1^ band corresponds to the characteristic absorption of peptide bonds, particularly the carbonyl (C = O) stretch. Additional bands at 1536 and 1239 cm^−1^ were attributed to amide II and III (a II / a III), respectively.^[^ ^39^ ^]^ The control showed maximum intensity due to the unaltered gelatin structure. Chemical modifications affected the secondary structure of gelatin, altering the relative intensity (^*^) but not the position of amide I and II bands.

### 2.2 Swelling and Hydration Capacity

The swelling behavior of the four compositions was investigated in both double‐distilled water (ddw) and PBS to understand their dimensional stability and liquid absorption capacities under different conditions (Figure 2B). In water, the GC‐CBX/AMN matrix exhibited the highest swelling (323%) due to its broad pore size distribution and mixed functionalization of graphenic species, which introduces numerous hydrophilic sites that enhance hydrogen bonding with water. Conversely, all the other formulations did not reach a water uptake ratio of more than 67%. The control showed the lowest swelling, suggesting, perhaps, that it exhibits the most stable, crosslinked structure, favored by the lack of graphenic sheets hindering the chemical crosslinking, which limits water uptake and expansion. GC‐AMN also displayed low swelling but with slightly higher values due to amino groups that promote hydrogen bonding, while GC‐CBX showed somewhat more enhanced swelling due to its carboxyl groups and broader pore distribution, which increases water absorption relative to the control and GC‐AMN matrices.

In PBS, the profiles of liquid uptake (Figure 2B, dotted curves) are much more similar and vary less than in the case of water, with very similar profiles and a range of liquid uptake situated between 110% and 210%. The GC, GC‐AMN, and GC‐CBX matrices swelled more in PBS than in ddw, indicating stronger interactions with ionic components and a superior fluid mobility supported by the ionic strength of the medium.

The GC‐CBX matrix displayed the greatest swelling because of strong interactions between carboxyl groups and ions, which significantly enhanced water absorption. In contrast, GC‐AMN showed the lowest swelling due to its hydrophobic aliphatic chains and weaker affinity for ions. GC‐CBX/AMN matrix demonstrated a substantial but moderate swelling degree, as the presence of –(CH_2_)– chains dampens its interaction with ions. Also, its swelling kinetics first resemble the slower profile of GC‐AMN but continue in the latter part of the timeframe to reach values comparable to GC‐CBX.

Overall, the kinetic analysis revealed that the GC matrix absorbed liquid quickly initially but then stabilized. GC‐AMN showed minimal and slow swelling overall, indicating a more hydrophobic character of the network, while CG‐CBX had an abrupt initial swelling followed by gradual stabilization. A unique structural behavior emerged in the case of GC‐CBX/AMN, which features an unaligned behavior that comes from the fact that it is the only nanocomposite that engulfs more ddw than PBS. That might be due to the opposite charge functionalization of the rGO fillers, which in ddw are rather passive (neutralized), but in an electrolytic environment, the ionic content is attained to drive electrostatic attraction and network densification. The ionic dynamics could be a key factor in the dimensional stability of the material, crucial for a proper host integration following implantation. Together, these results highlight how pore size, functionalization, and ionic interactions govern the distinct swelling profiles of these matrices in ddw versus PBS.

### 2.3 Degradation Kinetics and Scaffold Resorption

Figure 2C illustrates the evolution of gel fraction over time for the four samples. Initially, they all feature the same important weigh loss in the first hours of incubation; also, the three nanocomposites display a faster degradation rate compared to the GC composition, which can be correlated to the crosslinking hindering effect the planar nanofillers might have on the polymer network. Their degradation profile slightly fluctuates in time (as shown in the insets of the figure) but harmonizes toward the second half of the timeframe. All formulations stabilize at similar gel fraction values (25.1 ± 0.8%) after 14 days of incubation in collagenase solution, indicating comparable resistance to enzymatic degradation following an initial rapid degradation phase. At a weight loss of ≈75% of the t_0_ mass, we can speculate that the fraction of gelatin of the blend is fully degraded under collagenase activity.

### 2.4 In Vitro Biocompatibility Assays

**Figure 3:**
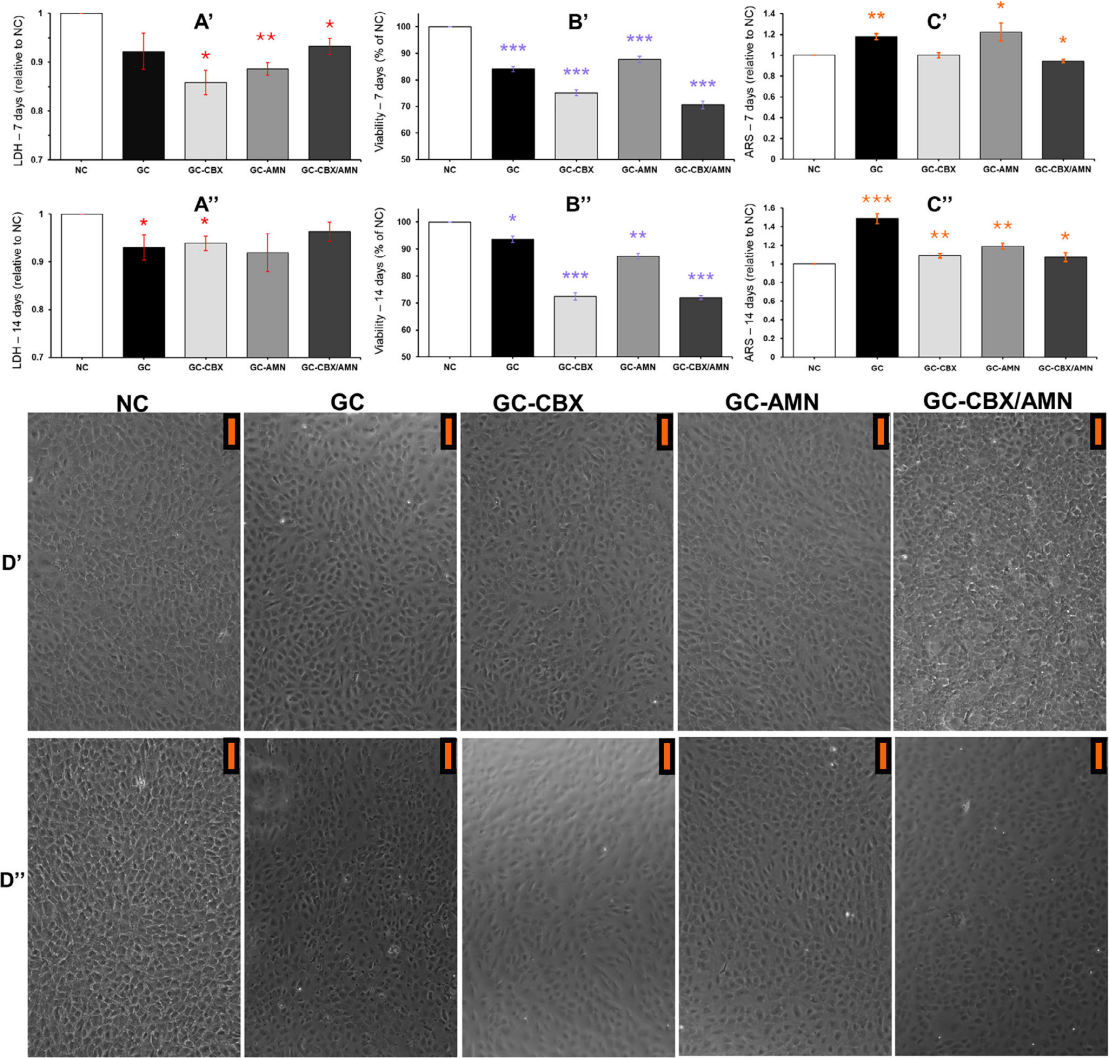
Cell viability, cytotoxicity, osteogenic differentiation, and morphology over 7 and 14 days. A) Lactate dehydrogenase (LDH) release (relative to NC), B) metabolic activity (MTT assay), and C) mineralized matrix deposition (Alizarin Red staining) were assessed for MC3T3‐E1 cells cultured on the tested materials at 7 days (A′–C′) and 14 days (A″–C″). D) Representative microscopy images of MC3T3‐E1 cells at 7 days (D’ panels) and 14 days (D” panels). Upper corner orange scalebar represents 100 µm.

The cellular response of MC3T3‐E1 pre‐osteoblasts to GC‐type nanocomposite scaffolds was assessed at 7 and 14 days using MTT and LDH assays, Alizarin Red staining, and optical microscopy (**Figure** **3**). These evaluations collectively provided insights into cellular viability, metabolic behavior, membrane integrity, and osteogenic differentiation.

After 7 days of direct scaffold–cell contact, all tested materials maintained viability levels above the cytocompatibility threshold of 70% (Figure 3B′), confirming their biocompatibility. The GC‐AMN composite exhibited the highest cell viability (87.6 ± 1.2%), followed by GC (84.1 ± 0.9%), with both showing statistically significant increases relative to the negative control (*p* < 0.001). Incorporation of rGO‐CBX led to reduced metabolic activity: GC‐CBX showed a ≈9% decrease in viability compared to GC (*p* < 0.001), while GC‐CBX/AMN decreased by ≈17% relative to GC‐AMN (*p* < 0.001). At 14 days (Figure 3B′’), this trend persisted: GC and GC‐AMN maintained the highest MTT signals, while GC‐CBX scaffolds showed significantly reduced values compared to their respective counterparts without rGO‐CBX, yet all remained above the cytocompatibility threshold.

The LDH assay revealed minimal extracellular enzyme release across all conditions at both timepoints. At 7 days (Figure 3A′), slight elevations in LDH levels were noted in the GC and GC‐CBX/AMN groups, yet these remained close to baseline and statistically insignificant in terms of cytotoxicity. At 14 days (Figure 3A’″), LDH values across all samples remained low and consistent with untreated controls, suggesting the preservation of membrane integrity and absence of lytic damage under all scaffold conditions.

Mineralization analysis via Alizarin Red staining demonstrated early and progressive osteogenic differentiation in response to all scaffolds. At 7 days (Figure 3C′), GG and GC‐AMN composites promoted the strongest calcium deposition, with fold increases of ≈1.18 and 1.31, respectively, over untreated controls (*p* < 0.01). GC‐CBX showed slightly lower mineralization, yet still significantly above control levels (*p* < 0.05). After 14 days (Figure 3C″), the GC scaffold exhibited the highest mineralization (1.5–1.6 × control, *p* < 0.001), followed by GC‐AMN. While CBX composites remained less potent in promoting in vitro mineralization, they still maintained statistically significant osteoinductive activity compared to the negative control (*p* < 0.05).

Optical microscopy images acquired at 7 (Figure 3D′) and 14 days (Figure 3D″’) revealed that cells maintained a typical polygonal morphology with extensive surface coverage in all scaffold conditions. The monolayers were dense and showed signs of over‐confluence, consistent with healthy adhesion and proliferation. No significant morphological abnormalities, detachment, or debris were observed. At 14 days, all groups exhibited comparable confluency, with GC and GC‐AMN groups appearing slightly more uniform and densely populated.

### 2.5 Evaluation of Osteogenic Potential in Ectopic Implantation Models

#### 2.5.1 Clinical Observation of Experimental Animals

The mice exhibited no postoperative complications. Additionally, no mortality occurred following implantation, and no inflammation or infection was observed in the affected areas throughout the 8‐week experimental period.

#### 2.5.2 Biocompatibility and Immune Response

**Figure 4:**
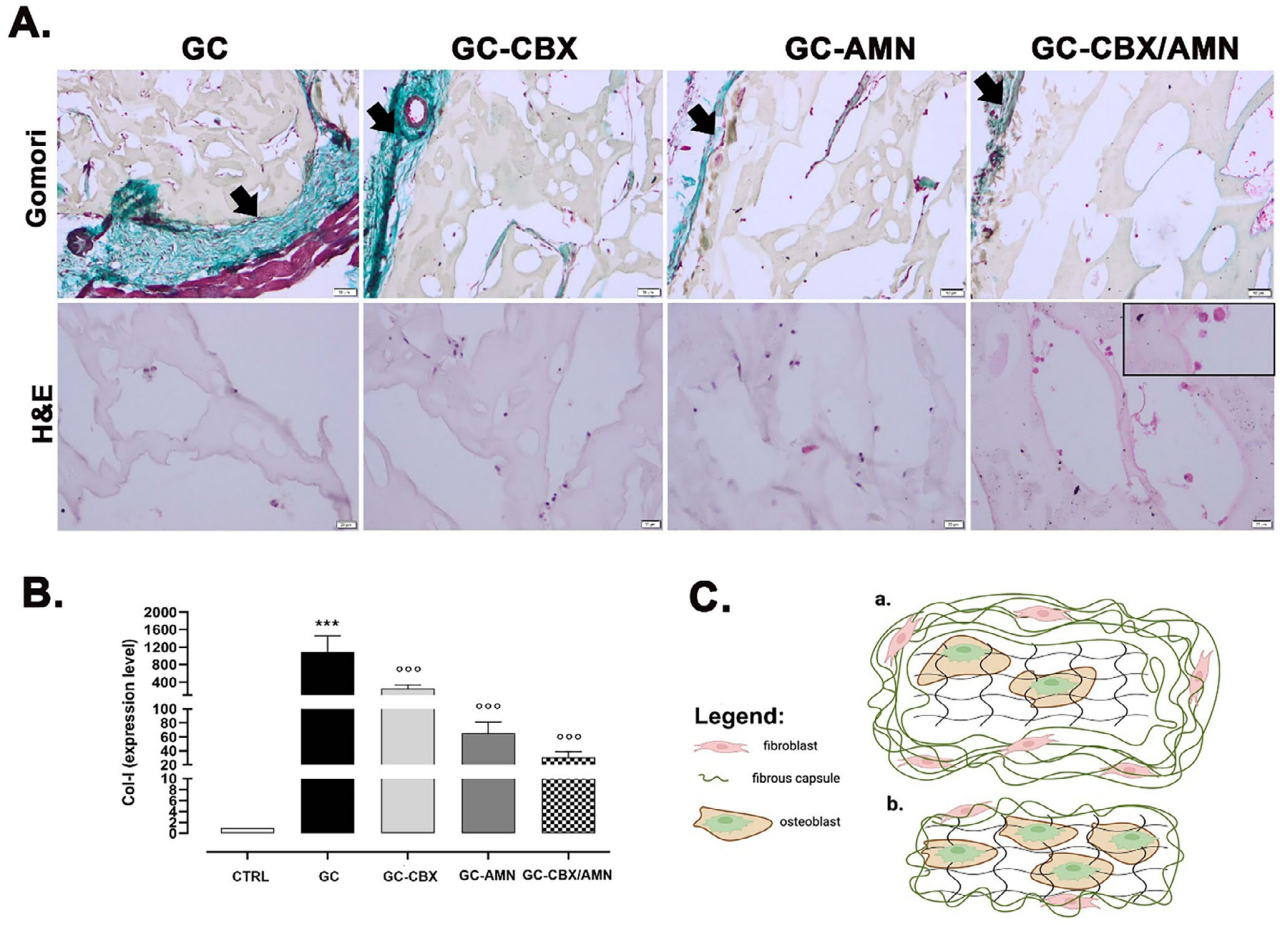
In vivo biocompatibility analysis of subcutaneous implants of the nanocomposites at 8 weeks post‐implantation. A) Gomori‐stained histological images showing (green) the variable thickness of the capsules (arrow) surrounding the different materials (50 µm scale bar) and the histological appearance of cell‐infiltrated materials stained with HE (20 µm scale bar). Detail – differentiated cells secreting matrix. B) Col‐1 gene expression for the experimental groups. ^***^ *p* < 0.001 versus CTRL, °°° *p* < 0.001 versus GC. C) Schematic representation of the implanted materials, highlighting the thick fibrous capsule with active collagen‐producing fibroblasts for GC and few differentiated osteoblasts in the scaffold (a), as well as a reduced capsule with few fibroblasts for GC‐CBX/AMN and more differentiated osteoblasts in the scaffold (b).

Gomori trichrome staining revealed the formation of a fibrous collagen capsule around the materials 8 weeks after subcutaneous implantation (**Figure** **4**A), particularly for GC, which explains the significant up‐regulation of Col‐1 for this group compared to the control. In contrast, for GC‐CBX, GC‐AMN, and GC‐CBX/AMN, the expression was significantly lower compared to GC. No foreign body giant cells (FBGCs) were observed in any of the groups at week 8.

#### 2.5.3 Ectopic Osteogenesis and Mineralization

**Figure 5:**
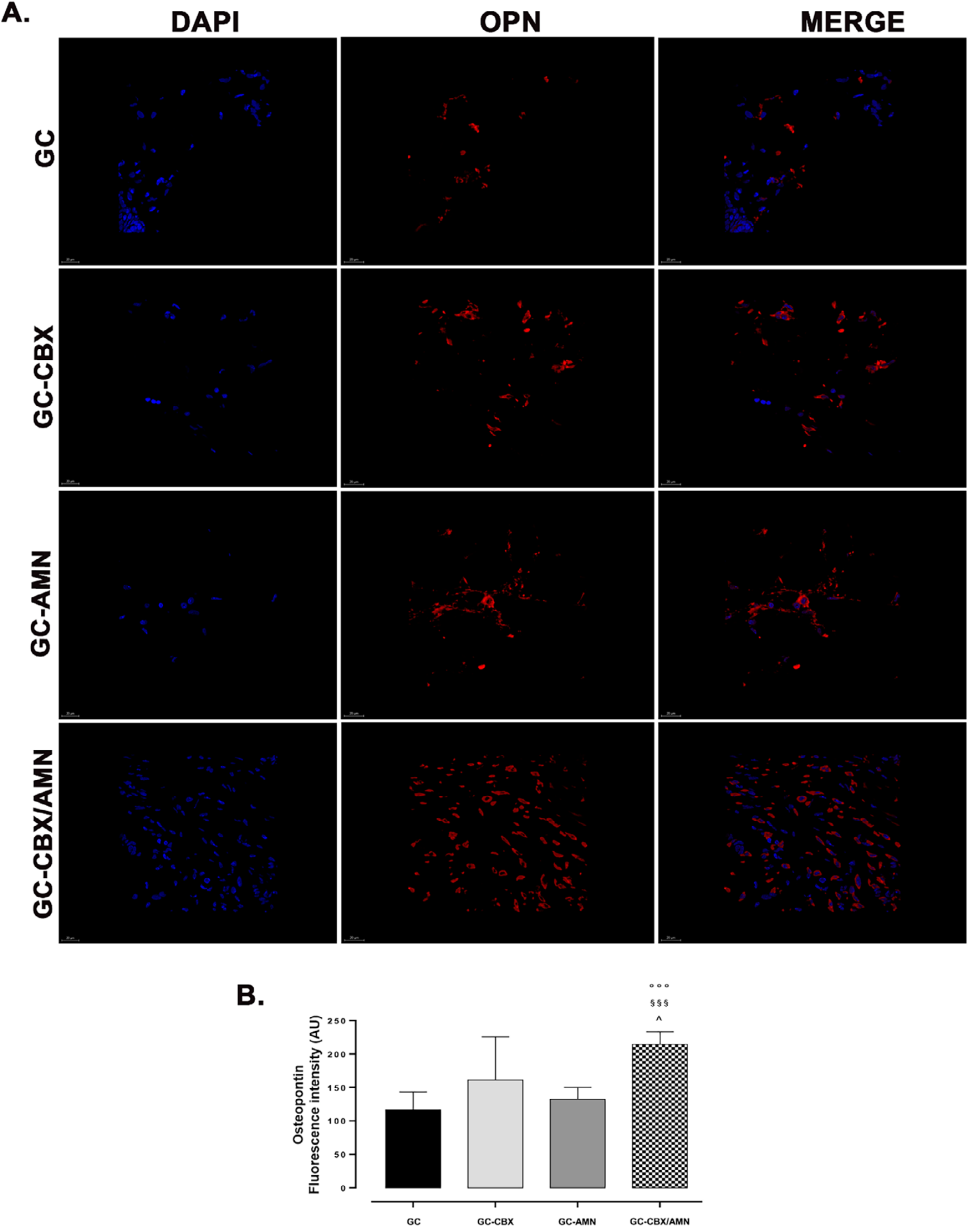
A) Immunofluorescence images showing OPN expression in subcutaneous implants at 8 weeks post‐implantation. IF‐red; DAPI nuclei (blue), Ob.63x.; B) Bar graphs showing semi‐quantification of fluorescence intensity for OPN. Data are presented as the mean percentage relative to control±SEM (*n* = 5); °°° *p* < 0.001 versus GC, §§§ *p* < 0.001 versus GC‐AMN; ^ *p* < 0.05 versus GC‐CBX.

**Figure 6:**
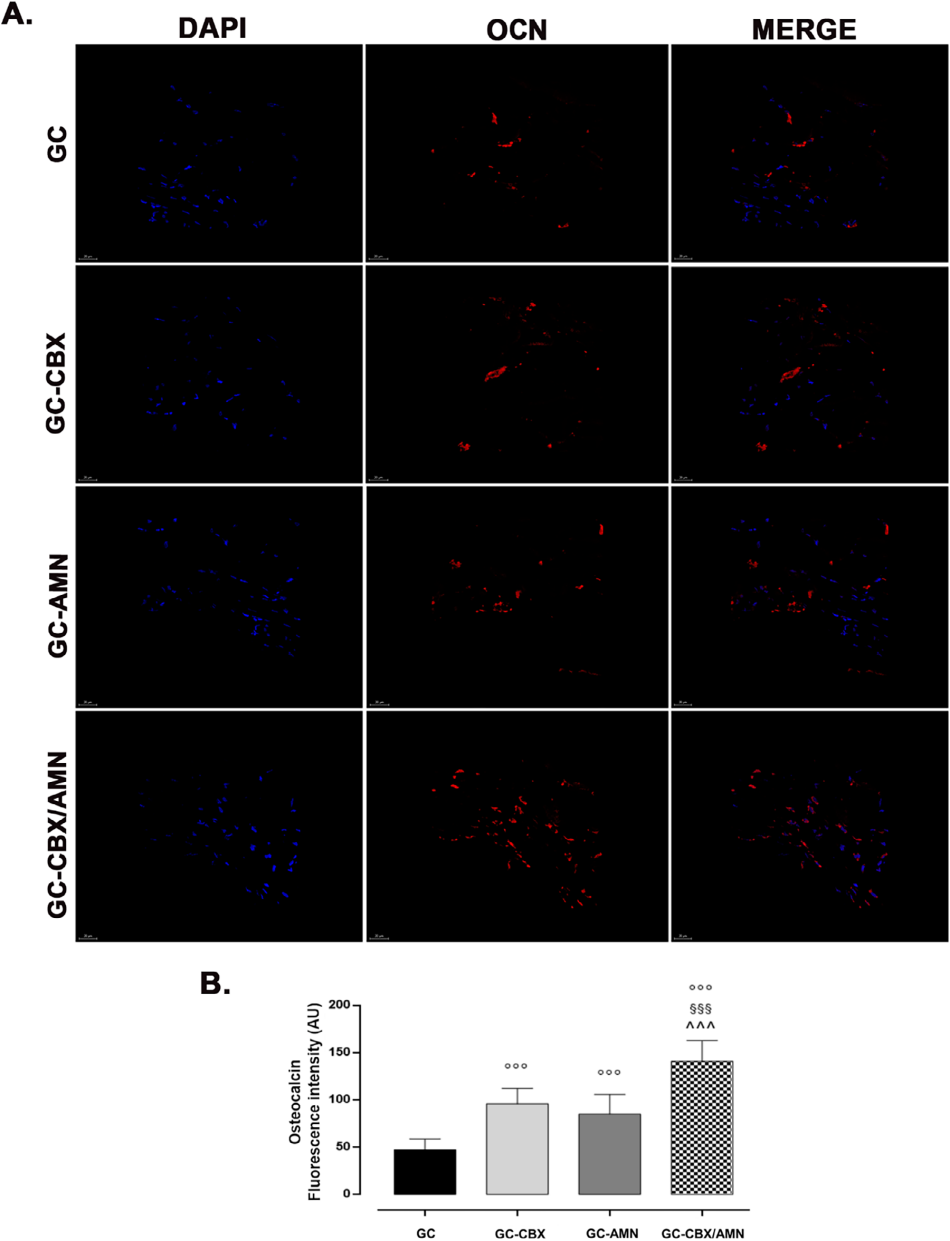
A) Immunofluorescence images showing OCN expression in subcutaneous implants at 8 weeks post‐implantation. IF‐red; DAPI nuclei (blue), Ob.63x.; B) Bar graphs showing semi‐quantification of fluorescence intensity for OCN. Data are presented as the mean percentage relative to control ± SEM (*n* = 5); °°° *p* < 0.001 versus GC, §§§ *p* < 0.001 versus GC‐AMN; ^ *p* < 0.05 and ^^^ *p* < 0.001 versus GC‐CBX.

To evaluate the osteoinductive potential of the implanted scaffolds in ectopic environments, we analyzed the expression of osteogenic markers osteopontin (OPN) and osteocalcin (OCN) by immunofluorescence (**Figures** **5**A and **6**A). All implanted scaffolds induced the expression of OPN, with notably lower levels of OCN. The highest immunopositivity for both markers was observed in the GC‐CBX/AMN group.

**Figure 7:**
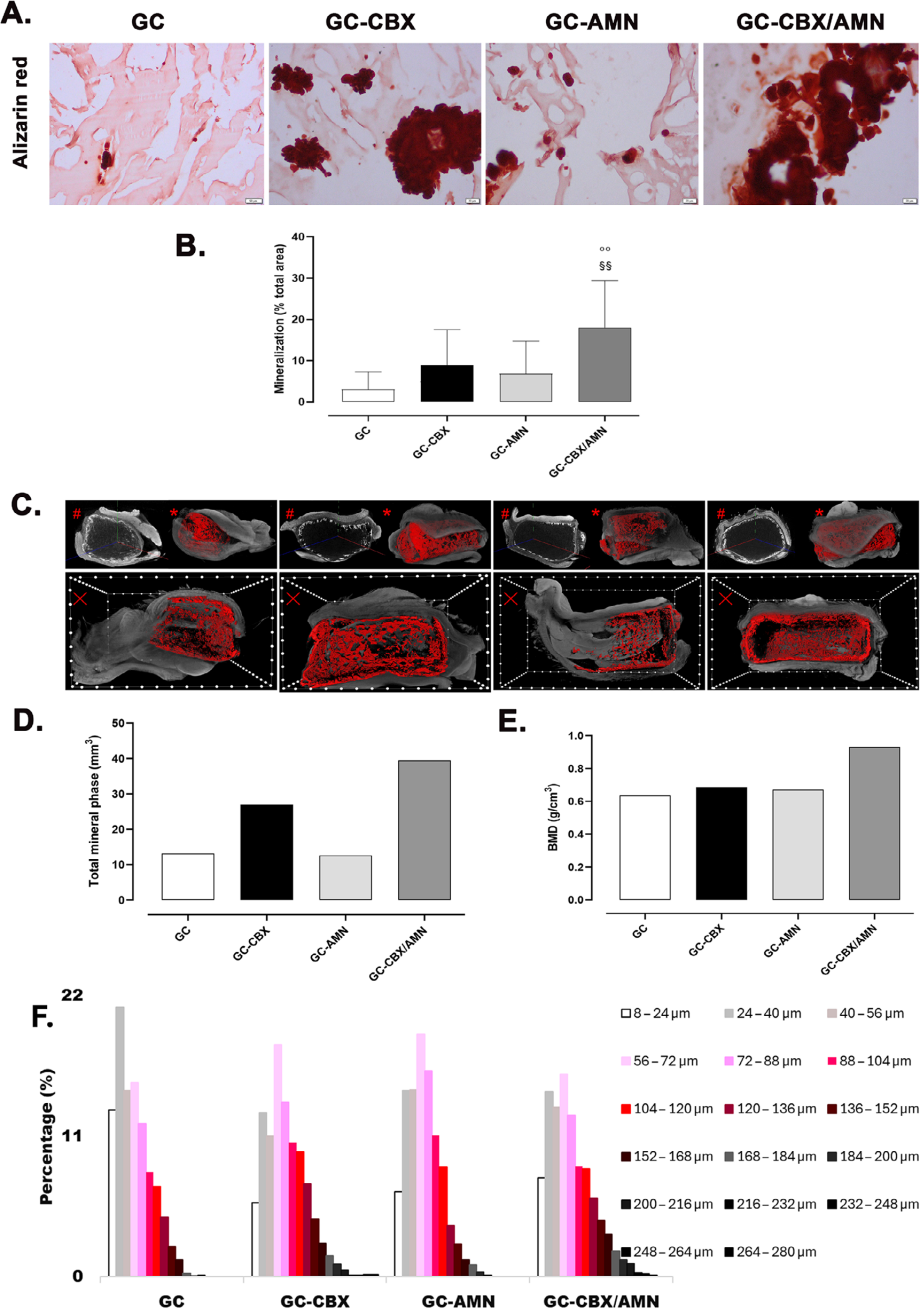
Analysis of the mineralization degree of subcutaneous implants at 8 weeks post‐implantation. A) Alizarin red staining (20 µm scale bar). B) Quantification of the degree of mineralization (% of the total analyzed area). °°° *p* < 0.001 versus GC, ^^^ *p* < 0.001 versus GC‐CBX, §§ *p* < 0.01 versus GC‐AMN. C) Color‐enhanced µCT images of scaffolds taken 8 weeks after implantation. D) Quantification of mineral volume. E) Bone mineral density measurements. F) Histogram analysis of mineral particle diameter distribution according to their maximum cross‐sectional diameter, reflecting material‐dependent differences in mineral nucleation and growth.

Matrix mineralization induced by osteoblasts was analyzed using Alizarin red staining. All materials showed mineralization clusters, more pronounced toward the edge of the implanted materials. Quantification revealed a significant increase in mineralization for the GC‐AMN and GC‐CBX/AMN materials compared to GC (**Figure** **7**). The total mineral phase and bone mineral density (BMD) varied among the analyzed samples, revealing distinct patterns in mineralization. GC and GC‐AMN exhibited similar total mineral phase volumes (13.1 and 12.6 mm^3^, respectively), while GC‐CBX showed a substantial increase to 27 mm^3^. The highest mineral phase content was observed in GC‐CBX/AMN, reaching 39.4 mm^3^. Despite this variation in mineral volume, BMD values remained relatively similar across GC, GC‐CBX, and GC‐AMN, ranging between 0.64 and 0.69 g cm^−3^. However, GC‐CBX/AMN displayed a significantly higher BMD of 0.93 g cm^−3^, suggesting a distinct effect when CBX and AMN were combined. These findings suggest that increased mineral content does not always correspond to proportional increases in mineral density, highlighting the distinct behavior of hybrid formulations.

## 3 Discussion

Nanocomposite scaffolds incorporating functionalized reduced graphene oxide derivatives, cellulose nanofibers, and hydrogel matrices represent a versatile and promising platform for bone tissue engineering, particularly for stimulating osteogenesis in ectopic environments. Recent advancements indicate that graphene‐based materials possess the potential to improve mechanical performance as well as osteoinductive capacity, thereby facilitating cell adhesion, proliferation, and differentiation.^[^ ^4^, ^40^ ^]^ Considering these findings, we undertook the development and evaluation of a series of innovative nanocomposite hydrogels, with particular emphasis on their structural characteristics, biological interactions, and degradation behaviors. Among them, the dual‐functionalized GC‐CBX/AMN formulation demonstrated exceptional performance, highlighting the promise of synergistic benefits of surface modification promoting scaffold‐guided bone regeneration.

Micro‐computed tomography conducted post‐synthesis but prior to implantation revealed that the scaffolds, particularly GC‐CBX/AMN, exhibited highly porous and interconnected architectures with optimal pore size distributions. These structural characteristics are consistent with the requirements for bone regeneration. Pores exceeding 300 µm are widely acknowledged as essential for cell migration, vascularization, and osteogenesis, supporting bone regeneration.^[^ ^41^ ^]^ Furthermore, the improved pore distribution in GC‐CBX/AMN scaffolds aligns with previous findings, where porous architectures facilitated rapid cell infiltration and bone matrix deposition.^[^ ^42^ ^]^ In our study, these large pores are interconnected through a network of evenly distributed smaller pores (shown in blue and violet, Figure 1), positioning these scaffolds as promising options for in vivo osteogenic studies, highlighting the potential of these scaffolds to support osteoblastic differentiation and tissue integration.

From a structural point of view, by FTIR, the nanocomposites exhibit typical characteristics of the original GC matrix, with intense O─H and C─H vibrations and well‐defined amides. However, even the low content of rGO‐AMN and rGO‐CBX was able to generate some interesting interphase interactions of electrostatic nature O─H, COO^−^ and amide regions. These interactions suggest electrostatic effects that contribute to the restructuring of the GC matrix, resulting in a more ordered and aligned molecular arrangement.

Also, a III signal is best defined in GC due to the unimpeded genipin crosslinking while the presence of nanofillers enlarges the absorption band and decreases the sharpness of the peak. rGO nanoparticles, due to carbonyl and amine groups can interact with pendant carboxyl and amine from the GC matrix components and form hydrogen bonds leading to a more ordered and stable structure; in particular for gelatin, peptide chains conformation can be altered since graphenic species, due to their unique electronic structure, can induce stronger degree of charge separation, have been proven to act as nucleating agents and to promote the alignment of gelatin chains and intramolecular hydrogen bonding.^[^ ^43^, ^44^ ^]^

This is also reflected in the C─O stretching vibrational peak at ≈1030 cm^−1^ that increased in intensity with the addition of rGO species because of their *π*‐electron system and electronic structure of the GC matrix. Apart from the additional C─O groups imparted by the nanofiller in the GC‐CBX and GC‐CBX/AMN compositions, a similar behavior appears in GC‐AMN that is attributed to increased order that facilitates the intense vibrational modes (#). The peaks from 1480–1358 cm^−1^ reflect the presence of carboxylate groups (symmetric COO^−^ stretching).^[^ ^45^ ^]^ Strong absorption in the control suggested carboxyl groups in the matrix. Compositing with modified reduced graphene oxide introduced changes in this band's absorption, in particular to the relative ratio of 1410/1451 cm^−1^ absorbance peaks (↔).^[^ ^46^ ^]^ The rGO nanoparticles intercalated between polymer chains resulted in increased interchain spacing leading to superior vibrational modes for the carboxylates of the polymer matrix that shifted the ration from sub‐unitary to over‐unitary.

The presence of dual‐functionalized rGO enhanced pore uniformity and broadened pore size distributions, providing pathways for cell proliferation, angiogenesis, and nutrient transport, essential for osteogenic activity.^[^ ^47^, ^48^, ^49^ ^]^ The dual functionalization of rGO with amino and carboxyl groups likely promoted electrostatic and hydrogen bonding interactions, stabilizing the scaffold network during gelation and lyophilization.^[^ ^48^ ^]^

The changes noted in the vibrational modes of the amide and carboxylate groups, along with the increased intensity of C─O and O─H interactions, indicate a greater level of molecular organization and interfacial bonding within the GC matrix. The structural adjustments that arise from the interactions between functionalised rGO and biopolymeric components play a crucial role in stabilising the hydrogel network. These adjustments are vital for enhancing protein adsorption, facilitating cell adhesion, and promoting the differentiation of progenitor cells. The specific alignment of gelatin peptide chains, coupled with an increase in interchain spacing, builds a biomimetic environment that closely resembles the native ECM, which is recognised for its influence on osteoblast behavior. Furthermore, the strengthening of hydrogen bonds and electrostatic interactions contributes to the resilience of the material throughout processing and implantation. This preservation is crucial for maintaining the porous architecture and the biochemical signals that are vital for both the initiation and sustenance of the osteogenic process.

The inclusion of CNFs and functionalized rGO markedly enhanced the mechanical integrity of the scaffolds. The GC‐CBX/AMN scaffolds demonstrated the greatest wall thickness and structural stability among the tested formulations, crucial for applications in load‐bearing environments.^[^ ^50^ ^]^ The reinforcing effect of CNFs, known for their high tensile strength and hydrophilicity, contributed to this improvement.^[^ ^51^ ^]^ Furthermore, functionalized rGO's ability to form strong interfacial bonds with the hydrogel matrix added mechanical resilience and reduced brittleness.^[^ ^52^, ^53^ ^]^

Swelling studies showed a significant influence of rGO functionalization on the hydrophilicity and hydration capacity of the scaffolds. The GC‐CBX/AMN formulation exhibited higher swelling in ddw, attributed to its broad pore distribution and functional groups promoting water uptake. This characteristic aligns with other findings, which showed that functionalized graphene increases hydrophilicity and enhances hydrogel swelling behavior.^[^ ^54^, ^55^ ^]^ In PBS, the swelling profiles were more uniform, suggesting a balancing effect of ionic interactions. These results underscore the importance of scaffold hydration in supporting cell attachment, proliferation, and differentiation, particularly in the early stages of implantation. Hydrated scaffolds provide a conducive microenvironment for progenitor cells and growth factor diffusion, facilitating osteogenesis.^[^ ^46^ ^]^

Controlled degradation is a critical attribute of scaffolds for bone tissue engineering, ensuring structural support during early tissue formation while allowing gradual resorption. The enzymatic degradation studies revealed that GC‐CBX/AMN scaffolds exhibited a biphasic degradation profile, with rapid initial mass loss followed by stabilization at ≈25% residual mass. This behavior aligns with the natural timeline of bone healing, providing sufficient structural support while facilitating cellular ingrowth and vascularization.^[^ ^56^, ^57^ ^]^

The faster degradation of rGO‐containing scaffolds compared to the control matrix may be attributed to the disruption of crosslinking by planar nanofillers, as observed in similar studies. Gelatin‐derived bioactive peptides released during degradation likely further enhanced osteogenesis by promoting the recruitment and differentiation of osteoprogenitor cells.^[^ ^10^ ^]^ In the context of nutrient flow and early‐stage vascularization, increased porosity resulting from scaffold degradation can enhance the diffusion of nutrients and oxygen, as well as the infiltration of cells into the scaffold structure. This degradation‐driven increase in porosity supports tissue integration and the formation of vascular networks, essential for the regeneration and sustenance of new tissue.^[^ ^42^ ^]^ Moreover, the controlled degradation of scaffolds not only promotes nutrient flow and vascularization but also ensures a gradual transition to long‐term stability. As the scaffold degrades, it shifts the mechanical load‐bearing responsibility to the maturing bone tissue. This allows more durable scaffold components to maintain structural support while the bone tissue strengthens and adapts to the increasing mechanical demands. This dynamic interplay is critical for effective bone regeneration and ensures that the scaffold supports tissue maturation without premature failure or hindrance to the natural growth process.^[^ ^58^ ^]^

These findings validate the cytocompatibility and osteoinductive potential of GC‐type nanocomposites in interaction with pre‐osteoblast cells. All evaluated scaffolds preserved viability above safety thresholds and promoted both metabolic activity and differentiation over a 14‐day culture period. The MTT and LDH assays (Figure 3A,B) together demonstrated that although scaffold composition can influence cellular behavior, none of the materials tested adversely affected membrane integrity or induced cytotoxic responses.

The increased MTT activity observed in GC and GC‐AMN scaffolds suggests that the synergistic combination of gelatin and aminated graphene components creates an optimal microenvironment for early cell adhesion and metabolic engagement. The reduction in MTT values noted in scaffolds containing rGO‐CBX, particularly in GC‐CBX/AMN, likely reflects altered material–cell interactions rather than outright toxicity. This hypothesis is reforced by the LDH results, which indicate no evidence of membrane disruption, and by preserved cellular morphology, showing structurally intact confluent cell layers.

Interestingly, the mineralization outcomes diverge slightly from the viability trends. While GC‐AMN showed the highest early metabolic activity, the GC scaffold alone drove the most robust differentiation at 14 days. This decoupling between cellular metabolism and differentiation outcome suggests that the physicochemical properties of the GC scaffold may offer stronger osteoinductive cues at later stages, even when initial cell growth is modest. These cues may include ionic interactions, surface topography, or substrate stiffness that preferentially stimulate matrix mineralization.

CBX‐containing scaffolds, although less supportive of early metabolic activity, did not inhibit differentiation. This suggests that CBX may moderate cell proliferation and metabolism without impairing lineage commitment or mineral deposition. Such modulation could be especially beneficial in contexts where controlled cell behavior or inflammation is a concern, such as in preventing excessive proliferation.

The preserved polygonal morphology and confluence in all samples (Figure 3D′,D″) further validate these findings, indicating that none of the materials exerted disruptive or stress‐inducing effects on cytoskeletal organization or adhesion.

Collectively, the GC and GC‐AMN scaffolds demonstrate strong potential to support both pre‐osteoblast viability and differentiation, while CBX‐functionalized materials may offer tunable biological effects that could be harnessed in context‐specific regenerative applications. These insights underscore the need for continued investigation into the underlying mechanisms governing scaffold‐cell interaction, particularly with regard to long‐term extracellular matrix development and in vivo efficacy.

Histological assessments validated the biocompatibility of all scaffold variants, with no evidence of inflammatory infiltrates or foreign body giant cells (FBGCs) in the subcutaneous implantation model. These findings are in agreement with previous studies reporting that graphene‐based scaffolds, particularly when functionalized with amino or carboxyl groups, exhibit low cytotoxicity and are well tolerated in vivo.^[^ ^59^, ^60^, ^61^ ^]^ The immunofluorescence analysis revealed a clear upregulation of osteogenic markers in all scaffold groups, with the most pronounced signal in the GC‐CBX/AMN condition, emphasising the synergistic impact of integrated functionalisation of rGO species.^[^ ^62^ ^]^ This is consistent with previous studies demonstrating that graphene derivatives can upregulate osteogenic gene expression via BMP‐2/Runx2 pathways, further promoting calcium accumulation on scaffold surfaces.^[^ ^4^ ^]^

The relatively low OCN expression across all conditions suggests that, while osteogenic commitment was initiated, full differentiation into mature osteoblasts was not achieved in the ectopic setting. These immunofluorescence findings correlate with mineralization data. Alizarin Red staining showed mineral clusters predominantly located at the periphery of the scaffolds, with limited spatial distribution of calcified matrix.

These observations underline both the potential and the constraints of ectopic osteogenesis. The subcutaneous model, devoid of marrow‐derived progenitors, vascular inputs, or mechanical cues, is a rigorous test of the intrinsic osteoinductive capacity of scaffolds.^[^ ^63^, ^64^ ^]^ In this context, the GC‐CBX/AMN scaffold demonstrated superior performance compared to other groups, yet the partial nature of differentiation and mineralization highlights the need for orthotopic validation, where the physiological environment may support complete bone regeneration.

The enhanced osteogenic response in GC‐CBX/AMN likely results from the synergistic action of the amino and carboxyl functional groups, which are known to influence protein adsorption, surface charge, osteogenic signaling, and calcium deposition.^[^ ^59^, ^62^, ^65^, ^66^ ^]^ The synergistic effects of amino and carboxyl functional groups in GC‐CBX/AMN scaffolds likely enhance these osteoinductive properties, further supported by their porous architecture and bioactive degradation products.

The enhanced in vivo performance of GC‐CBX/AMN, despite its moderate in vitro metabolic activities, presumably indicates biofunctional interactions related to immunological regulation, vascular support, or scaffold‐driven differentiation that are not present in simplified monoculture tests. Full differentiation potential emerges most clearly in vivo, where the functional triad of host‐derived biological signals, uninterrupted immune system modulation of cell and material by products, and tailored material properties collectively – shape the regenerative microenvironment.

The composites exhibited moderate levels of LDH release and MTT values, indicating minimal cytotoxicity while facilitating cell adhesion and metabolic activity without compromising the cell membrane, a finding further supported by optical microscopy. The slightly increased LDH in GC‐CBX/AMN formulations may indicate minor, non‐lethal instabilities in the cell membrane due to material‐cell interactions, without compromising cellular function at this stage; mild cellular stress may arise as cells interact with the material, particularly in a static culture where scaffold degradation and surface chemistry remain unaltered under immune response conditions. This response was counter‐regulated in vivo, potentially due to pro‐regenerative immune signaling^[^ ^67^ ^]^ like mild reactive oxygen species^[^ ^68^, ^69^ ^]^ or damage‐associated molecular patterns,^[^ ^70^ ^]^ alongside angiogenic stimulation,^[^ ^69^, ^71^ ^]^ mechanical triggers,^[^ ^72^ ^]^ and stress‐induced mineralisation^[^ ^73^ ^]^ which balanced the results of the 2D cell assay.

The observed disconnect between total mineral phase volume and BMD (Figure 7E) suggests that the mineralization process is influenced by factors beyond merely the amount of mineral deposition. The similar BMD values for GC, GC‐CBX, and GC‐AMN indicate that the introduction of CBX or AMN individually does not substantially alter the density of the mineralized phase, despite variations in total mineral content. This suggests that the organization, distribution, and potential compaction of mineral deposits play a crucial role in defining BMD rather than just the quantity of mineral present. The markedly higher BMD in GC‐CBX/AMN highlights a potential synergistic effect between CBX and AMN, which may enhance both mineral deposition and densification. These findings imply that while individual graphenic additives may influence mineral accumulation, their combined effect may be necessary to achieve significant improvements in bone‐like material properties. These observations regarding the structural arrangement of the mineralized phase are also supported by the mineral nuclei size distributions measured in the tomograms and depicted in Figure 7F whereby GC and GC‐AMN feature the least expanded distribution and the highest share of mineral within the smaller domains.

These findings emphasise generally the need of strategic nanocomposite design to reach multifunctional scaffolds suited for bone regeneration. Although all formulations maintained biocompatibility and osteogenic activity to different degrees, dual‐functionalized GC‐CBX/AMN scaffolds routinely outperformed their single‐modified counterparts in terms of structural, biochemical, and in vivo behavior characteristics. The combination of carboxylated and aminated versions of rGO with CNFs and bioactive hydrogels produced scaffolds that can replicate natural bone morphology and accurately regulate cellular behavior and mineralised tissue development. Notably, the enhancement in both bone mineral density and mineral phase organization underscores the significance of surface chemical functionalisation in influencing the quality, rather than only the quantity, of freshly created bone tissue. Altogether, these results reinforce the therapeutic promise of chemically engineered graphene‐based systems and establish a foundation for future translational investigations in orthotopic defect models and under dynamic physiological loading conditions.

## 4 Conclusion

Bone tissue engineering requires biomaterials able to concurrently satisfy structural, biochemical, and functional needs to enable regeneration in damaged tissues. Designed to synergistically increase osteogenesis and scaffold performance, this work sought to solve these difficulties by producing complex nanocomposite hydrogels with the focus on addressing the relevance of surface‐functionalized reduced graphene oxide and a low‐ratio reinforcement of natural biopolymer blends with cellulose nanofibrils. By tailoring surface chemistry through carboxylated and aminated rGO variants, we achieved scaffolds with enhanced mechanical resilience, tunable degradation, and robust osteoinductive potential – validated through in vitro assays and ectopic in vivo implantation.

The proven capacity of GC‐CBX/AMN scaffolds to facilitate ectopic osteogenesis renders them viable candidates for addressing major clinical challenges, sub as critical‐sized bone defects and non‐union fractures, where conventional approaches often fail. Their tailorable degradation kinetics, excellent biocompatibility, and potent osteoinductive properties meet the core criteria for scaffold‐based regenerative strategies, establishing a robust foundation for bone repair in environments devoid of native osteogenic stimuli. This performance is further strengthened by the unique synergistic interaction between surface‐charge‐modulated rGO derivatives and the supportive hydrogel matrix, which collectively modulate and guide cell behavior toward osteogenic commitment.

The efficacy of these scaffolds in facilitating bone growth in an ectopic subcutaneous model underscores their potential as advanced platforms for bone regenerative medicine. Subsequent research should advance toward comprehensive in vivo models to thoroughly evaluate scaffold‐host bone integration, vascularization efficiency, and mechanical resilience under physiological loading. Considering the encouraging osteoinductive characteristics exhibited in this study, GC‐CBX/AMN scaffolds may serve as a novel option to address existing challenges in bone healing and the treatment of non‐union fractures.

## 5 Experimental Section

### Preparation and Functionalization of Graphene‐Based Nanocomposites

The exfoliation procedure for graphene materials – reduced graphene oxide functionalized with carboxyl – rGO‐CBX and amino – rGO‐AMN groups, as well as a mixture of the two carbonaceous species CBX/AMN, both purchased from ACS Materials (Pasadena CA, USA), was performed using a VCX 750 ultrasonic (Sonics & Materials, Inc.,53 Church Hill Road, Newton, CT 06470‐1614, USA). The sonic probe, equipped with a Ti‐6Al‐4 V tip, to a 750 W processor operating at a frequency of 20 kHz. The ultrasound oscillation amplitude was maintained at 70% throughout the 1 h long exfoliation. The process was conducted at a concentration of 1 mg mL^−1^, with the samples placed in ice‐cold water baths to prevent overheating. A comparative analysis of the features the carbonaceous species used in this study can be found in the Supporting Information file.

To prepare the nanocomposite matrix, 0.64 g of fish gelatin (Sigma–Aldrich–Gelatin from cold water fish skin, BioReagent) was dissolved in 1 mL of double‐distilled water (ddw) at ≈40 °C, with continuous stirring until fully dissolved. Separately, 0.16 g of gellan gum (Gellan gum, Sigma–Aldrich) was added to 2 mL of a graphene species dispersion (1 mg mL^−1^ graphene). This mixture was heated to 70 °C and stirred until the gellan gum was completely dissolved.

After its complete dissolution, 5 mL of carboxylated cellulose nanofibrils (CNFs) suspension (1.2% concentration) was gradually added to the gellan gum mixture at 70 °C, under continuous stirring to ensure homogeneity. During the process, the mixture was allowed to cool to 50 °C with sustained stirring to prevent the formation of non‐uniform gels.

CNFs were obtained by oxidizing never‐dried bleached kraft pulp from softwood (kindly provided by Stora Enso) using a TEMPO‐mediated process. This process produced gel‐like CNFs suspensions with a solid content of 1.2% and a modification degree of 835 µmol g^−1^, determined via conductometric titration.^[^ ^74^ ^]^

At 50 °C, the gelatin solution from the first step was incorporated into the CNF‐gellan mixture. Stirring was continued for 30 min to ensure thorough homogenization and allowed to cool at 40 °C. At this stage, 3.2 mg of genipin (Genipin≥98% (HPLC), was added to the homogeneous CNF‐gellan‐gelatin‐graphene derivative blend. The mixture was stirred continuously at 40 °C for 45 min to ensure uniform distribution of genipin.

The prepared nanocomposites were poured into 96‐well plates, covered, and incubated at 37 °C overnight to complete the cross‐linking process. After incubation, the plates were immersed in a calcium chloride (CaCl_2_, Sigma–Aldrich) bath solution for gellan crosslinking. Finally, the samples were frozen and freeze‐dried (−90 °C, 0.01 mbar, for 72 h).

The four nanocomposites and their corresponding designations are as follows: the control – gelatin‐gellan gum‐CNF → GC; gelatin‐gellan gum‐CNF/aminated reduced graphene oxide composition → GC‐AMN, gelatin‐gellan gum‐CNF/carboxylated reduced graphene oxide composition → GC‐CBX; gelatin‐gellan gum‐CNF/carboxylated and aminated reduced graphene oxide composition → GC‐CBX/AMN.

All the other regents used in the characterization process, collagenase (lyophilized powder from *Clostridium histolyticum*, ≥125 CDU/mg solid), sodium azide (≥99.5%), Tris‐HCl, Phosphate Buffer Saline (PBS) tablets and ethylenediaminetetraacetic acid (EDTA, ≥99%) were purchased from Sigma–Aldrich and used without further purification.

### Morphological Analysis by Micro‐Computed Tomography (µCT)

Micro‐computed tomography was performed using a high‐resolution Bruker µCT 1272 system. Pre‐implantation samples were scanned without a filter at 50 kV and 145 µA, with an exposure time of 350 ms per frame, a rotation of 180° at 0.2° steps, and 3‐frame averaging. The pixel size was set to 7 µm. Explanted samples were scanned with a 0.25 mm Al filter, at 75 kV and 37 µA, with 1400 ms exposure, 360° rotation at 0.3° steps, and 3‐frame averaging, with an image pixel size of 8 µm. All scans were performed using a 2452 × 1640 pixel detector. 3D reconstruction of tomograms for all samples was performed using Bruker NRecon 1.7.1.6 software (Kontich, Belgium). Quantitative analysis and generation of an inverted dataset, where pores are reconstructed as solid objects, were conducted in Bruker CTAn 1.17.7.2 (Kontich, Belgium). The resulting solid and pore datasets were rendered (both separately and simultaneously for the video supplementary material) in Bruker CTVox 3.3.0.0 (Kontich, Belgium). BrukerCTAn software was used to analyze tomograms and measure the morphological parameters of the scanned samples (total porosity, pore/wall/mineral size distribution, etc.) and to generate a color‐coded secondary dataset illustrating wall thickness variations. All procedures were carried out after thresholding (binarization – white pixels for the solid sample, black pixels for pores) and despeckling (removal of residual scanning artifacts). Metric unit conversion was based on the image pixel size. Bone mineral density (BMD) was measured using reconstructed µCT images of the explants and X‐ray attenuation coefficients, calibrated with known densities (0.25 and 0.75 g cm^−3^) of hydroxyapatite phantoms. CTAn software (Bruker) was used to derive mean BMD values from ROIs in the scaffold's mineralised areas using consistent thresholding parameters.

### Structural Analysis by Fourier Transform Infrared Spectroscopy (FTIR)

FTIR measurements were conducted using a Bruker Vertex 70 FTIR spectrometer (USA) equipped with an ATR (Attenuated Total Reflectance) accessory. For each sample, 32 scans were recorded in ATR‐FTIR mode at room temperature, with a resolution of 4 cm^−1^ across a wavenumber range of 800–4000 cm^−1^.

### Swelling Degree

The swelling profile of the nanocomposite materials was evaluated by calculating the percentage weight change following immersion in distilled water and phosphate‐buffered saline (PBS) at 37 °C. Pre‐weighed dry samples (w_o_) were hydrated at predefined time intervals (t_x_), then removed from the incubation medium, blotted with filter paper to remove excess water, and reweighed (w_tj_). The swelling capacity (%) was calculated using the following equation:

(1) $$\Delta s\;\;\left[\%\right]=\frac{w_{tj}-w_{d}}{w_{d}}\times 100$$

where Δs represents the swelling degree, and w_tj_ is the mass of the samples at each measurement time. The maximum swelling percentage was monitored up to 72 h, both in distilled water and PBS. Based on 12 measurement times, the swelling kinetics were determined as follows:

(2) $$k_{j}=\frac{\Delta s_{j}}{t_{j}}$$

where k_j_ represents the swelling rate, Δs_j_ is the swelling degree at each time, and t_j_ is the time (in minutes).

### Stability Study in Simulated Physiological Media – Enzymatic Degradation

For the in vitro degradation study, samples were immersed in a collagenase solution (10 µg mL^−1^). The degradation medium consisted of distilled water containing 10 mm Tris‐HCl, 10 mm NaCl, 10 mm EDTA, and 10 mm NaN_3_. Scaffold degradation was monitored over a period of up to 3 weeks, with incubation at 37 °C in triplicate for each sample. Degradation was stopped by removing samples from the immersion medium at varying time intervals: 4, 8, 10, 12 hours, 1, 3, 5, 7, 14, and 21 days. Samples were then dried at 37 °C, and the gel fraction (GF) was determined using the following equation:

(3) $$GF\left(\%\right)=\left(\frac{W_{d,t}}{W_{0}}\right)\times 100\%$$

where*W*_*d*,*t*_ represents the mass of the sample after drying at each degradation time point t, and *W*_0_​ is the initial mass of the sample. The evolution of the gel fraction was monitored up to 21 days. Based on the 10 measurement intervals, degradation kinetics were assessed:

(4) $$k_{GFj}=\frac{\Delta \mathrm{GF}{\left(\%\right)}_{j}}{t_{j}}$$

where k_GFj_ represents the degradation kinetics, ΔGF(%)_j_ is the gel fraction at each measurement time, and t_j_ is the time (in hours).

### In Vitro Biocompatibility and Osteogenic Potential Assessment

To assess biocompatibility and osteogenic potential, MC3T3 osteoblast precursor cells (ATCC, MA, Virginia, USA) were cultured in Eagle's Minimal Essential Medium (EMEM, Biochrom Merck, Darmstadt, Germany) supplemented with 10% fetal bovine serum (FBS, Biochrom Merck, Darmstadt, Germany) and 1% Penicillin – Streptomycin (P/S, Biochrom Merck, Darmstadt, Germany), under standard incubation conditions (37 °C, 5% CO2, 90% humidity).

Cells were seeded at a density of 100 000 cells/well in 24‐well plates to allow cell attachment. Meanwhile, scaffold samples were sterilized via ultraviolet (UV) exposure. Following the samples sterilization, these were placed onto the pre‐osteoblast cells, in direct contact, and incubated for 7 and 14 days under the same standard culture conditions.

### In Vitro Biocompatibility and Osteogenic Potential Assessment_Cell Viability

Cell viability was assessed using the MTT tetrazolium salt assay. Following the incubation period, the cell culture medium was removed from each well and replaced with 10% MTT solution in EMEM. Cells were incubated 1 h to allow MTT to be metabolized into formazan. The resulting formazan crystals were solubilized in DMSO, and absorbance was measured at 570 nm. Cell viability was calculated relative to the negative control (untreated cells), which was assigned a reference viability of 100%.

### In Vitro Biocompatibility and Osteogenic Potential Assessment_Cytotoxicity

Cytotoxicity was investigated by measuring the Lactate dehydrogenase (LDH) in the extracellular medium of each sample, using the CyQUANT LDH Cytotoxicity Assay (Invitrogen, Thermo Fisher Scientific, Waltham, Massachusetts, USA). For each sample, 50 µL of supernatant were collected after incubation and mixed with the reaction substrate. After a 30 min incubation, the stop solution was added, and the absorbance was measured at 490 nm. LDH release was expressed realtive to the negative control.

### In Vitro Biocompatibility and Osteogenic Potential Assessment_Cell Differentiation and Mineralization

Cellular differentiation was evaluated via calcium mineral deposition using Alizarin Red S staining. After 7 and 14 days of exposure to biomaterials, cells were washed with phosphate‐buffered saline (PBS) and fixed in 4% paraformaldehyde overnight at 4 °C. Cells were then stained with 40 mm Alizarin Red S (Sigma–Aldrich, Merck, Darmstadt, Germany) for 1 h, followed by multiple washes with deionized water until the supernatant remained clear. To quantify mineralization, cells were incubated with 10% acetic acid (Sigma–Aldrich) for 1 h at room temperature. The extract was transferred to microcentrifuge tubes, heated at 80 °C for 10 min, cooled, and neutralized with an equal volume of 10% ammonium hydroxide. Absorbance was measured at 405 nm, and mineralization was expressed relative to the negative control.

### In Vitro Biocompatibility and Osteogenic Potential Assessment_Cell Morphology

The cell morphology was investigated using optical microscopy, with no prior preparation of the pre‐osteoblast cells.

Data was expressed as mean ± STDEV (standard deviation) and the statistical evaluation was performed using Student t‐test, where ^*^ *p*<0,05, ^**^ *p*<0,01, respectively ^***^ *p*<0001.

### In Vivo Experimental Protocol

For this study, male CD1 mice aged 6–8 weeks and weighing 20–30 g were acquired from the Animal Facility of Vasile Goldis Western University. All animal handling adhered to EU Directive 2010/63/EU, with approval from the University's Scientific Research Ethics Committee (Approval No. 27/31.05.2023) and authorization from the National Veterinary and Food Safety Authority (ANSVSA Authorization No. 23 of 13.07.2023).

The mice were housed in individually ventilated cages with free access to food and water, maintained at standard conditions of 22 ± 2 °C, 50 ± 5% relative humidity, and a 12 h light/dark cycle. Surgical procedures were performed under aseptic conditions, with the mice anesthetized using ketamine/xylazine.

The four previously described materials were hydrated for 1 h prior to implantation into subcutaneous pockets on the dorsal side of mice (*n* = 10 per group). A separate control group of animals without implants was also included. Eight weeks post‐implantation, tissue samples were collected under anesthesia and processed for histopathological, molecular, and µCT imaging analyses.

### Histology Assays

After harvesting, the explants were fixed in 10% neutral‐buffered formalin for 24 h, followed by dehydration through increasing ethanol concentrations (70%, 80%, 90%, and 100%), clearing in xylene, and embedding in paraffin. Tissue sections, 5 µm thick, were cut and mounted on glass slides.

For H&E staining, the sections were deparaffinized, rehydrated through a graded ethanol series, and immersed in hematoxylin to stain the nuclei, followed by eosin to stain the cytoplasm. After dehydration, the sections were coverslipped for microscopic examination.

For Gomori trichrome staining, the sections were deparaffinized, rehydrated, and treated with Weigert's hematoxylin to stain the nuclei, followed by Gomori's trichrome solution to differentiate tissue components. Collagen and mineralized bone appeared green, while cytoplasmic elements were stained red. The sections were dehydrated and coverslipped with mounting medium.

Matrix mineralization was assessed using Alizarin Red staining to evaluate osteoblast‐mediated mineral deposition. The percentage of the mineralized area was quantified by analyzing 10 images per slide at 20x magnification.

The stained sections were examined using an Olympus BX43 microscope equipped with an Olympus XC30 digital camera.

### Immunofluorescence

Paraffin‐embedded tissue sections (5 µm thick) were deparaffinized and rehydrated using a graded ethanol series. Heat‐induced epitope retrieval (HIER) was performed, after which the slides were treated with 2% BSA to block nonspecific binding. The sections were then incubated at room temperature for 2 h with primary antibodies against osteocalcin (OCN) and osteopontin (OPN), diluted 1:200. Detection of the primary antibodies was achieved by applying a secondary antibody conjugated with Alexa 594. The slides were analyzed using a Leica TCS SP8 multispectral confocal laser scanning microscope. The fluorescence intensity of OPN and OCN was analyzed using ImageJ64 software (NIH, Bethesda, Maryland, USA). For each sample, five fields were randomly selected exclusively within scaffold regions exhibiting evident cellular infiltration, as identified by DAPI nuclear staining. Quantitative analysis was based on the mean fluorescence intensity per field and was expressed in arbitrary units (arb. units).

### Statistical Analysis

The obtained data were statistically evaluated using one‐way ANOVA, followed by Tukey's multiple comparison test. GraphPad Prism 6.0 software (San Diego, CA, USA) was used for this analysis. All results are presented as mean ± SD from *n* = 3 experiments, and *p*‐values < 0.05 were considered statistically significant.

### Declaration of Generative AI and AI‐Assisted Technologies in the Writing Process

During the preparation of this manuscript, George Mihail Vlăsceanu used ChatGPT (GPT‐4, OpenAI; model last updated April 2024) to assist in the writing process. The tool was used to improve the language, clarity, and coherence of the manuscript (e.g., including refining phrasing and drafting figure captions). At no point was the AI used to generate scientific content or data interpretation. The author carefully reviewed, edited, and verified all AI‐assisted content to ensure factual accuracy and alignment with the research. The author takes full responsibility for the final content of the manuscript.

## Conflict of Interest

The authors declare no conflict of interest.

## Supporting information



Supporting Information

## Data Availability

The data that support the findings of this study are available on request from the corresponding author. The data are not publicly available due to privacy or ethical restrictions.
