# Supplementary material for: Functionalized Reduced Graphene Oxide‐Based Nanocomposite Hydrogels for Enhanced Osteogenesis in Bone Tissue Engineering
Source: Adv Healthc Mater. 2025 Aug 7;14(31):e01941. doi: 10.1002/adhm.202501941 (PMC12683214; doi:10.1002/adhm.202501941)
Supplement: Supplementary file 1 — Supporting Information [file ADHM-14-0-s001.docx]

**Supplementary material for Functionalized Reduced Graphene Oxide-Based Nanocomposite Hydrogels for Enhanced Osteogenesis in Bone Tissue Engineering**

**Materials and methods**

Carboxylated reduced graphene oxide (rGO-CBX) and aminated poly(ethylene glycol) functionalized reduced graphene oxide (rGO-AMN) from ACS Materials were used in this study. Exfoliation procedure was carried out in double distilled water. UV-Vis spectroscopy was performed on liquid dispersion while the rest of the analyses on the solid content dried after sonication.

The exfoliation procedure involved a particular amount of rGO species to be dispersed in double distilled water to produce the dispersions at the concentration of 1mg/ml. The ultrasonic process was carried out utilizing Sonics & Materials, Inc.'s (53 Church Hill Road, Newton, CT 06470-1614 USA) VCX750 equipment for small and medium volumes, which was fitted with a titanium alloy probe tip (Ti -6Al-4V) and a 750 W source operating at a frequency of 20kHz. The probe's vibration amplitude was set to 70 %. The dispersions were obtained on ice bath. The process duration was varied varied from 20 – 40 – 60 minutes for the samples characterized by UV-Vis spectroscopy and kept to 60 minutes for the rest of the analyses. For all, the ultrasound pulse to pause ratio was fixed at 10 to 5 seconds.

*UV-vis spectrophotometry*

UV–vis absorbance of graphenic dispersions was measured on a Agilent Cary 60 UV-Vis Spectrophotometer equipment provided with a quartz cell having a light path of 1 mm. The initial dispersion (1mg/ml) was diluted to a ratio of 1:25 and the analyzed volume was 0.2 µl per sample.

*Fourier Transform Infrared Spectroscopy (FTIR)*

FTIR analysis was performed using a BRUKER VERTEX 70 spectrometer. The range used was 4000-500 cm^-1^, the number of scans is 32 and the resolution is 4 cm^-1^. The samples were analyzed from KBr pellets.

*X-Ray Diffraction (XRD)*

XRD analysis was performed on a X’Pert PRO MPD Panalytical equipment, in the range 2θ = 2–60°. An X-ray beam characteristic to Cu Kα radiation was used (λ = 1.5418 Å). Interlayer spacing (d-value) was calculated according to Bragg's law ^[1]^ :

d= λ/(2*sin(θ)), Eq. S1,

grain size (C) was calculated using the Scherrer equation^[2]^ :

C= (0.9* λ)/( β*cos(θ)), Eq. S2

whereby β is the Full Width at Half Maximum of the peak and the number of graphene sheets^[3]^ with:

n = C/d, Eq. S3.

*Raman Spectroscopy (RS)*

Raman analysis were performed with a Renishaw inVia Raman confocal spectrometer, using a 473 nm laser excitation (Renishaw, Brno-Černovic, Czech Republic), the 100× objective, and 5% laser power.

*X-ray Photoelectron Spectroscopy (XPS)*

The XPS analysis was performed with a K-Alpha device from Thermo Scientific equipped with an anode monochromatic Al K_α_ source (1486.6 eV) at a pressure of 2x10-9 mbar.

*Transmission Electron Microscopy (TEM)*

For morphological investigations of rGO-CBX and rGO-AMN specimens, further dilutions of the 1 mg/mL graphene dispersions to a concentration of 0.1 µg/mL were performed; 50 µL of the diluted dispersion were dropcasted onto a carbon-coated copper TEM grid support (Christine Gröpl, Tulln, Germany) and left undisturbed for solvent evaporation. After drying, independent samples were characterized with a TECNAI F30 G2STWIN equipment.

**Results**

UV–Vis spectrophotometry was employed to evaluate the exfoliation and dispersion efficiency of functionalized rGO species (Fig. S1) over various sonication intervals. All spectra exhibited characteristic π–π* transitions in the 220–300 nm range^[4]^, consistent with 2D carbon materials.
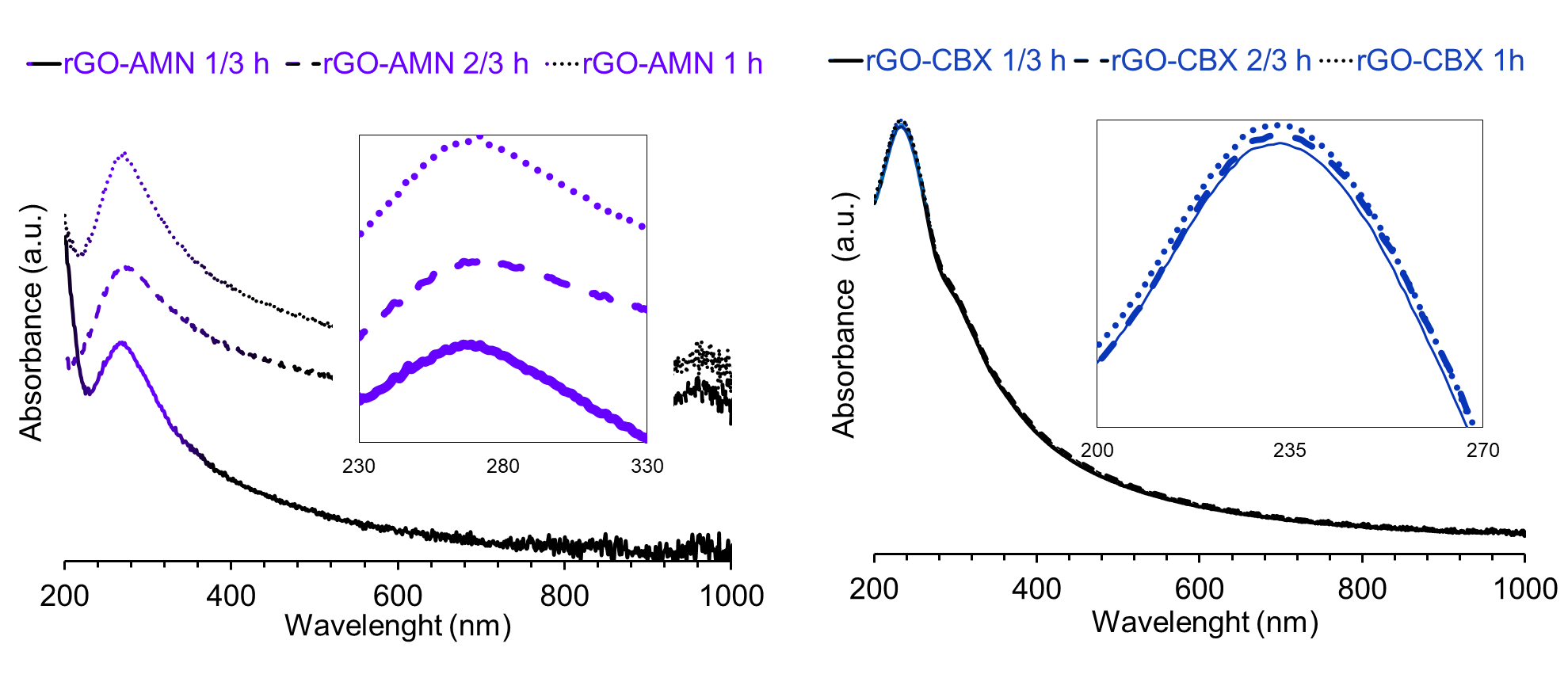


Figure S1. UV-Vis spectra of rGO-AMN and rGO-CBX

For both materials, absorbance increased progressively with sonication time, peaking at 1 h for rGO-AMN (λ ≈ 271 nm) and rGO-CMX (λ ≈ 233 nm). These maxima indicate improved exfoliation and dispersion. Notably, rGO-CBX showed a stronger and more defined absorption profile, suggesting a higher degree of exfoliation.^[5]^ In contrast, rGO-AMN displayed broader peaks and wavelength shifts, likely due to structural perturbations introduced by the PEG-modified amine functional groups.

Overall, the spectra confirm that sonication times influence differently the rGO species, but ensure the attainment of promising dispersions, validating the selected processing parameters for composite formulation.

FTIR spectroscopy was utilised to examine the chemical structure and functional group availability of rGO-CBX and rGO-AMN after ultrasonication. The comparative study of unprocessed (0 h) and sonicated (1 h) samples facilitates the evaluation of exfoliation efficiency and the spectrum progression of essential functional groups. Particular focus was directed towards alterations in distinctive vibrational bands linked to carboxyl, amine, and graphitic domains, as these variations indicate both chemical modification and morphological reorganisation of the two-dimensional carbon framework and are critical for the interaction with the composite matrix and cells.


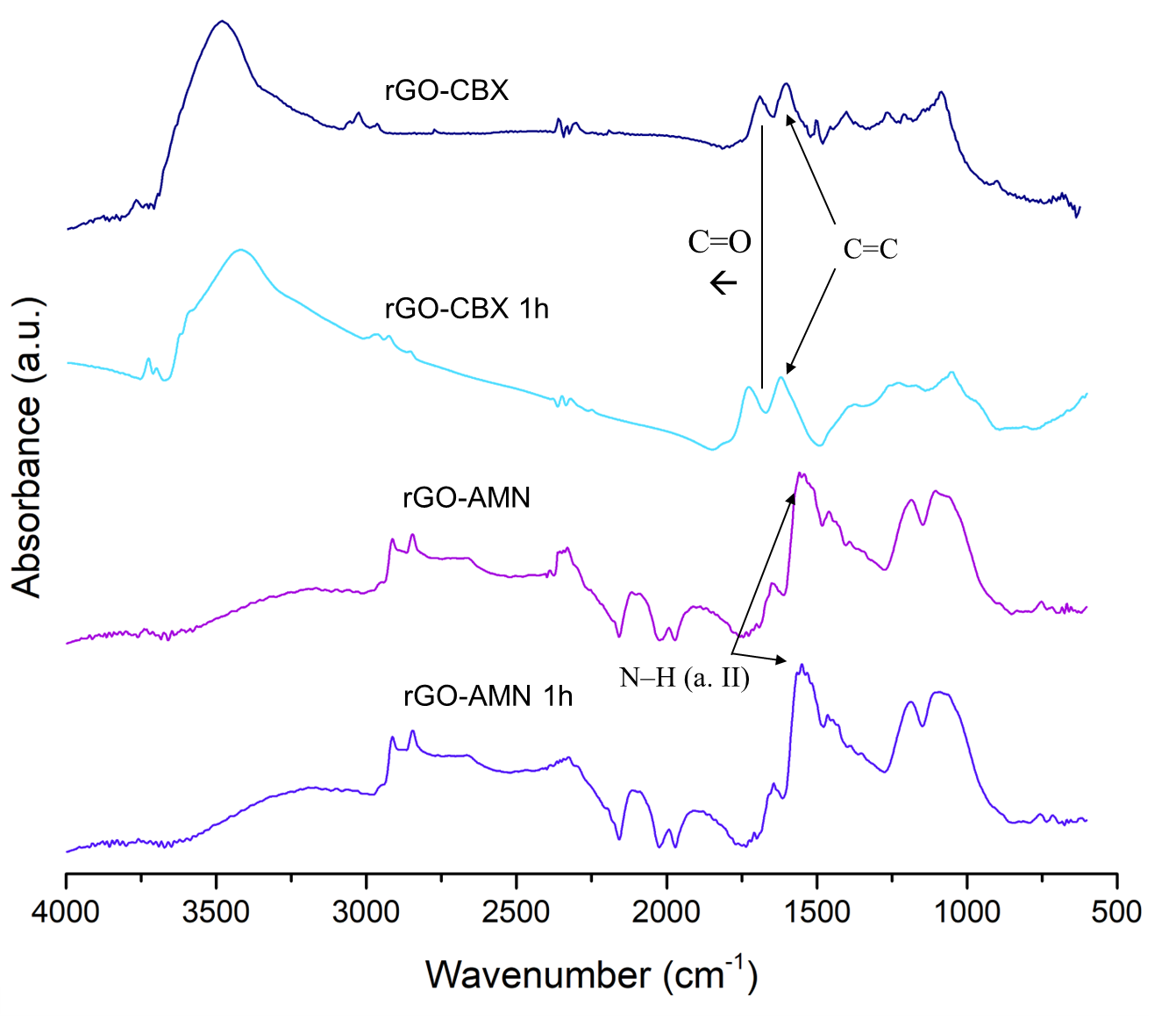


**Figure S2**. FTIR spectra of rGO-CBX and rGO-AMN before and after exfoliation

The FTIR spectra of rGO-CBX before and after one hour of sonication reveal significant alterations that signify enhanced exfoliation and increased accessibility of functional groups. A distinct peak at around 1720 cm⁻¹, indicative of the C=O stretching vibration of carboxylic acid groups, exhibits a subtle red shift and enhanced clarity in the 1 h sonicated sample, implying improved accessibility of surface –COOH groups resulting from sonication-induced delamination. The peak at approximately 1620 cm⁻¹, often attributed to C=C skeletal vibrations of sp² hybridised carbon domains, is more pronounced in the sonicated sample, suggesting partial restoration or unmasking of graphitic structures.^[6,7]^ A little blue shift is observed around 1200–1250 cm⁻¹, associated with C–O stretching vibrations, following sonication. This alteration may be ascribed to reorganisation at the sheet boundaries or increased contributions from surface oxygen groups that become more spectroscopically active as dispersion enhances.

The FTIR spectrum of amine-functionalized rGO (rGO-AMN) indicates alterations in conformational and chemical environments with respect to the N–H bending vibration (amide II band), initially centred at roughly 1560 cm⁻¹, experiences a significant red shift to around 1530–1540 cm⁻¹, suggesting modifications in hydrogen bonding or relaxation of the functional groups at the sheet surface.^[8]^ This transition aligns with sonication-enhanced exposure of PEG–amine groups and possible rearrangements at the polymer-functional group interface. The peak at around 1100–1150 cm⁻¹, linked to C–N or C–O stretching, becomes more pronounced after sonication, hence reinforcing the enhanced surface exposure of functional groups. A wider and somewhat more pronounced absorption band is aboserved about 3400 cm⁻¹ in the 1-hour sample, presumably due to N–H and/or O–H stretching vibrations.^[9]^ The augmented width and intensity of this peak indicate an improved hydrogen bonding capability, potentially resulting from higher chain mobility or the exposure of polar groups after sonication.

The combined spectrum alterations substantiate the assertion that both -CBX and -AMN functionalized rGO present enhanced exfoliation while also preserving the functionalisation, hence increasing the accessibility of both aromatic and oxygen-containing domains essential for composite interface.

X-ray diffraction (XRD) analysis was performed to investigate the structural evolution of rGO-CBX and rGO-AMN upon 1 h sonication. In both cases, exfoliation and partial disordering of the graphitic structure were observed. For rGO-CBX, the main (002) diffraction peak shifted from 2θ = 11.65° ^[9]^ to 11.16° (Figure S3), accompanied by a slight increase in interlayer spacing (from 0.759 to 0.792 nm) and a reduction in calculated sheet number (from 3 to 2), indicating improved exfoliation.^[10]^

Additionally, the emergence of new peaks at 40–41° post-sonication, absent in the untreated material, suggests the onset of turbostratic disorder – an enseble of randomly stacked or misaligned graphene layers ^[11]^. These structural changes align with enhanced dispersibility and surface accessibility of the carboxyl-functionalized rGO.


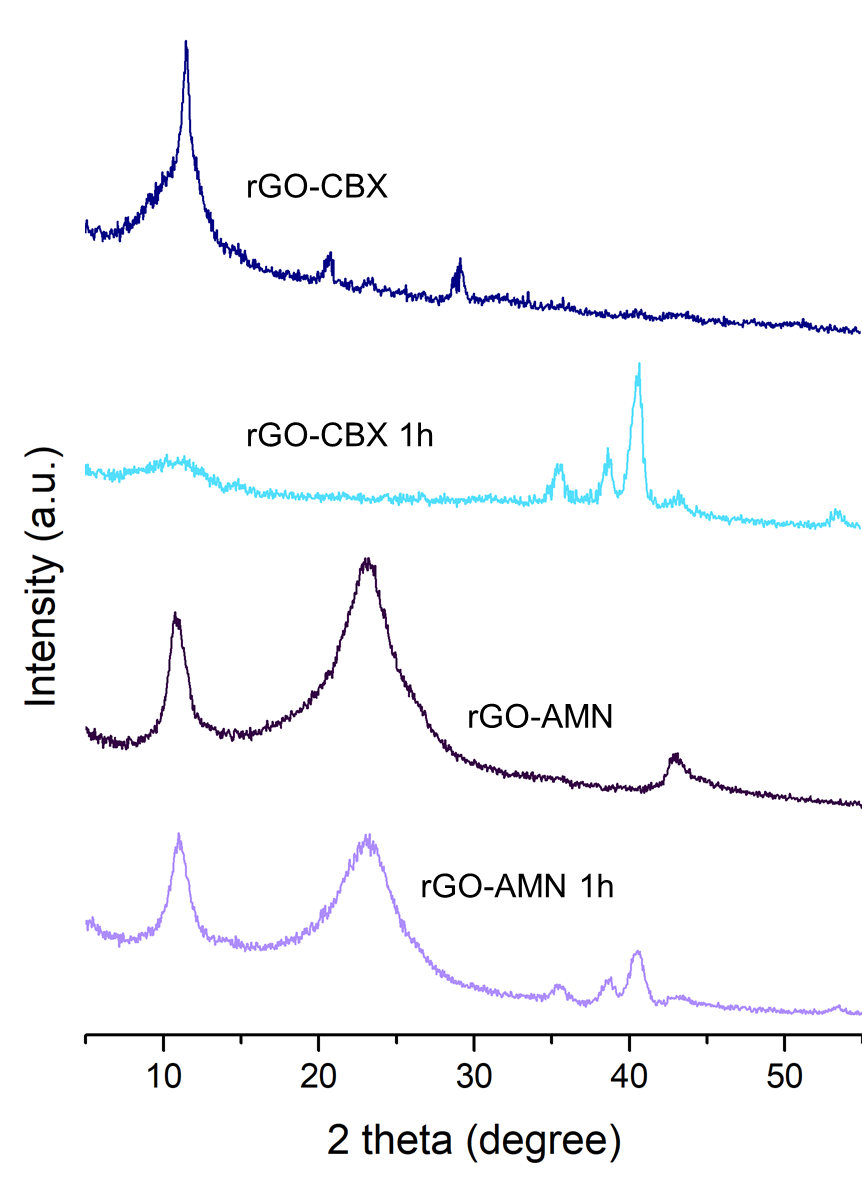


**Figure S3**. XRD of rGO-CBX and rGO-AMN before and after exfoliation

In the case of rGO-AMN, similar effects were observed, albeit less pronounced. The (002) reflection shifted from 10.77° to 10.98°, with a modest decrease in interlayer spacing (from 0.820 to 0.805 nm). Although the number of calculated stacked layers remained constant (n = 6), a visible broadening of the (002) peak and the appearance of diffraction features at ~40–43° also point to increased lattice disorder and partial delamination^[12,13]^. The relatively limited exfoliation efficiency in rGO-AMN may stem from steric hindrance/conformational constraints imposed by the PEG-linked amine moieties.^[1]^ Overall, the XRD results confirm that ultrasonication enhances the structural disassembly and disorder in both systems, more effectively in rGO-CBX, consistent with FTIR and UV–Vis findings and complete calculated data are detailed in Table S1 below.

Table S1. Analysis of relevant XRD peaks in the spectra of rGO-CBX and rGO-AMN before and after exfoliation

| **Sample** | **2θ [deg]** | **d-value [nm]** | **C [nm]** | **Number of sheets** | **2θ [deg]** | **Observation** | **2θ [deg]** | **Observation** |
| --- | --- | --- | --- | --- | --- | --- | --- | --- |
| rGO-CBX | 11.65 | 0.759 | 1.98 | 3 | 21.12 | Alteration of graphene reduction degree | - | Indication of "turbostratic disorder" within the carbon layers stacking^[11,12]^ |
| rGO-CBX - 1h | 11.16 | 0.792 | 1.77 | 2 | - |  | 40.71 |  |
| rGO-AMN | 10.77 | 0.820 | 5.21 | 6 | 23.2 |  | 43.09 |  |
| rGO-AMN - 1h | 10.98 | 0.805 | 4.63 | 6 | 23.43 |  | 40.51 |  |

Raman spectroscopy reveals key structural transformations in the rGO-CBX and rGO-AMN species following 1 hour of ultrasonication, highlighting variations in defect density, sp² domain ordering, and stacking configuration. Across all samples, characteristic D (~1345 cm⁻¹), G (~1595 cm⁻¹), and 2D (~2680–2720 cm⁻¹) bands were observed, with discernible 2D′ shoulders (Figure S4). The inset deconvolutions of the 2D and 2D′ regions further clarify the evolution of graphitic order. The intensity and area ratios (I(D)/I(G), A(D)/A(G), I(2D)/I(2D′), A(2D)/A(2D′)) are summarized in Table S2, indicating increased exfoliation and turbostratic disorder upon sonication, particularly for rGO-CBX.


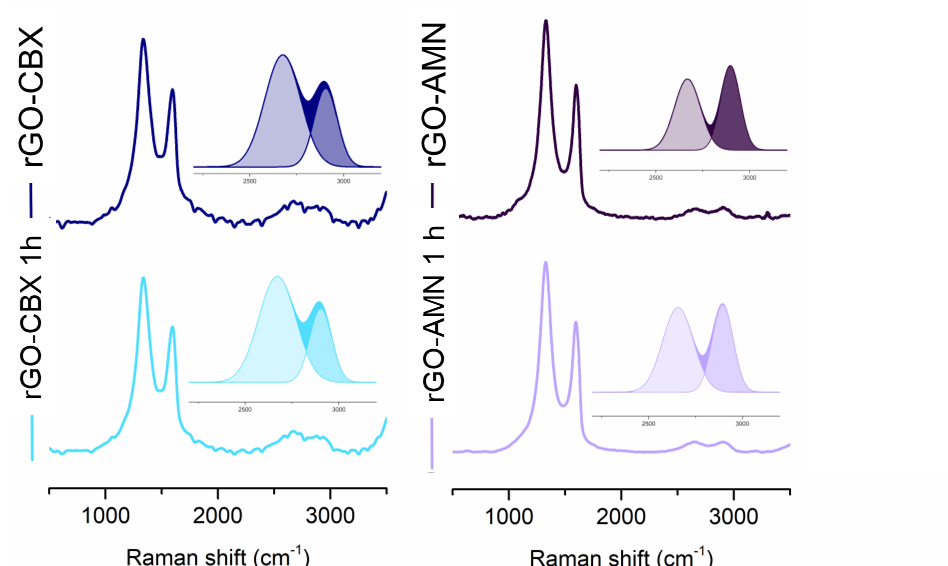


**Figure S4**. Raman spectra of rGO-CBX and rGO-AMN samples before and after 1 h ultrasonication, highlighting D, G, and 2D bands. Insets show deconvoluted 2D/2D′ regions to illustrate changes in stacking order and interlayer coupling.

For rGO-CBX, sonication increased the A(D)/A(G) ratio from 1.93 to 2.01 and I(D)/I(G) from 1.37 to 1.38, indicating a mild but consistent rise in defect density, likely from edge fragmentation or increased exposure of defect sites during exfoliation. More telling, however, is the rise in I(2D)/I(2D′) (1.056 → 1.095) and A(2D)/A(2D′) (2.20 → 2.43), suggesting a marked increase in interlayer decoupling and π–π disruption. These spectral shifts—also visible in the increased broadening and slight red shift of the 2D band—reflect enhanced delamination and turbostratic disorder, consistent with XRD data (d-spacing increase from 0.759 to 0.792 nm, and sheet number drop from 3 to 2). Taken together, these changes confirm that rGO-CBX responds strongly to ultrasonication, achieving greater exfoliation and more dynamic surface properties.^[14]^

**Table S2.** Raman intensity and area ratios for D, G, and 2D bands of rGO-CBX and rGO-AMN before and after 1 h sonication

| **Sample** | **A(2D/2D')** | **A(Id/Ig)** | **I(Id/Ig)** | **I(2D/2D')** |
| --- | --- | --- | --- | --- |
| rGO-AMN | 1.12 | 2.22 | 1.46 | 0.91 |
| rGO-AMN 1h | 1.42 | 2.16 | 1.45 | 1.00 |
| rGO-CBX | 2.20 | 1.93 | 1.37 | 1.06 |
| rGO-CBX 1h | 2.43 | 2.01 | 1.38 | 1.10 |

In contrast, rGO-AMN exhibited more modest spectral changes. The A(D)/A(G) and I(D)/I(G) ratios remained relatively stable (2.22 → 2.16 and 1.46 → 1.45, respectively), implying minimal change in defect density. However, the I(2D)/I(2D′) ratio increased from 0.91 to 1.00, and A(2D)/A(2D′) from 1.12 to 1.42, indicating some degree of electronic structure reorganization and partial decoupling. Still, the subtler changes compared to rGO-CBX suggest that PEG–amine functionalization imposes steric hindrance, limiting effective exfoliation and reordering.^[14,15]^ This is visually supported in the figure by the relatively conserved line shape of the 2D bands and the less prominent separation of 2D/2D′ peaks post-sonication.

XPS analysis (Figure S5, Table S3) demonstrates significant chemical evolution of the rGO-CBX and rGO-AMN materials upon 1 h sonication, particularly in terms of hybridization states and surface functionalities. In the case of rGO-CBX, the sp² C contribution increases from 3.12% to 3.9%, and the sp²/sp³ ratio shifts from 2.19 to 3.59, indicating partial regraphitization or confirming the exfoliation and increased contact surface of graphene domains.^[16]^ Concomitantly, oxygenated species such as hydroxyls and epoxides (C–OH / C–O–C) decrease, suggesting removal of labile oxygen groups. The total O1s area decreases along with the O/C ratio, consistent with mild deoxygenation. These data point toward sonication-driven defect healing and stabilization of π-conjugated domains, without significant formation of new redox-active groups.

Conversely, rGO-AMN undergoes more pronounced structural reorganization upon sonication. The sp² C content nearly doubles with the sp²/sp³ ratio increasing from 2.29 to 4.74, highlighting enhanced electronic delocalization. Interestingly, rGO-AMN samples exhibit substantial nitrogen incorporation – N1s signals rise from 6460.2 to 49093.5, with dominant increases in sp² N (4439.2 → 30273.7) and graphitic N, both known to support conductivity and catalytic potential^[17]^. The π–π* satellite also intensifies from 1425.1 to 2853.7, suggesting restoration of aromatic domains. Together, these features imply a synergistic restructuring where sonication facilitates N-doping and stabilization of redox-active oxygen moieties, distinguishing -AMN as a chemically enriched and electronically versatile rGO derivative.^[18]^


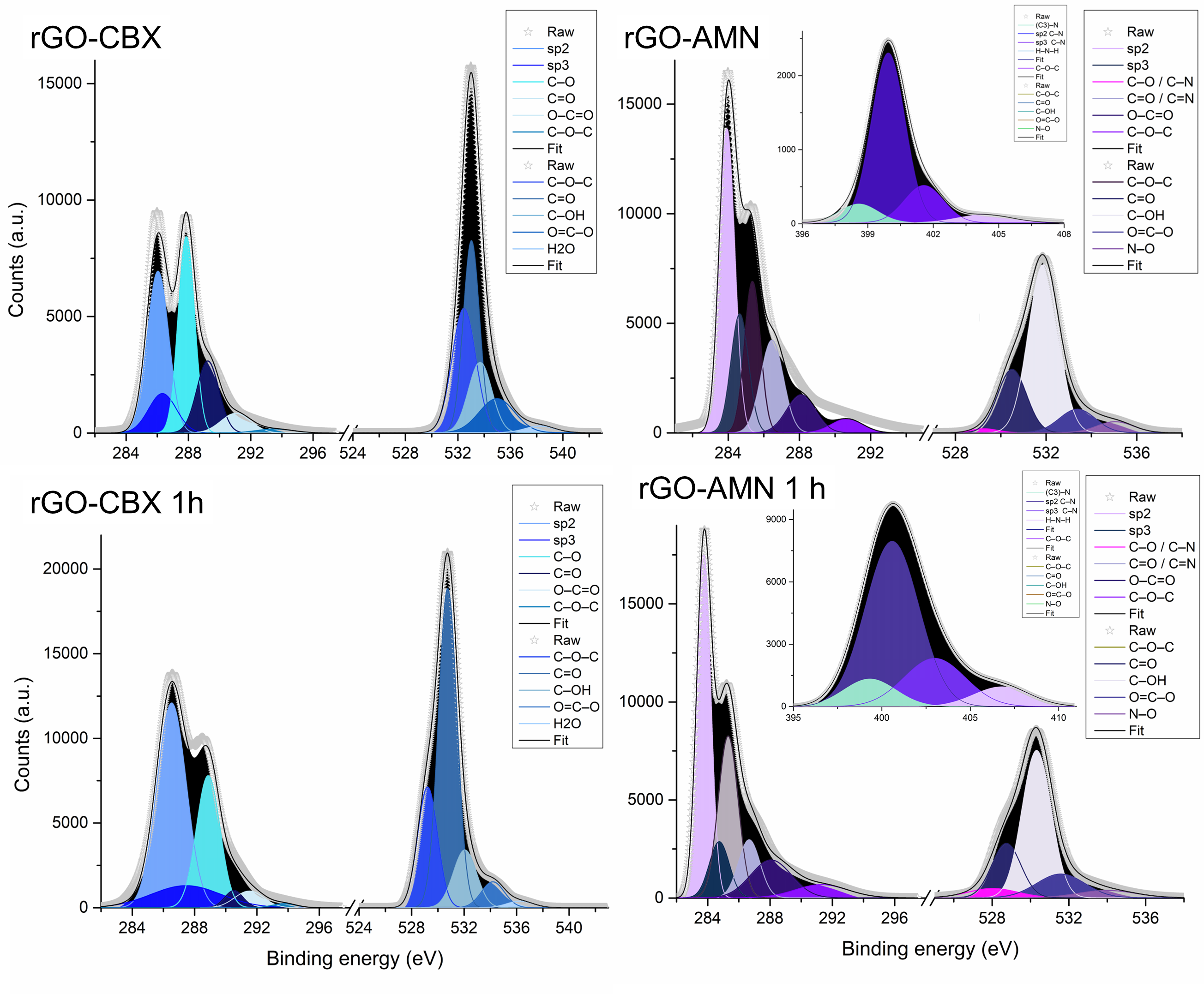


**Figure S5**. High-resolution XPS spectra (C 1s, O 1s, and N 1s) for rGO-CBX and rGO-AMN samples before and after 1 h of sonication.

Based on XPS deconvolution, distinct surface charge trends can be inferred for the functionalized rGO species following 1 h sonication. In the case of rGO-CBX, a reduction in surface oxygen content (O1s from 28.09% to 26.72%) and a marked decrease in hydroxyl/ether functionalities (C–OH/C–O–C from 1.18% to 0.52%) suggest a partial deoxygenation and regraphitization of the surface.^[19]^ These chemical changes imply a likely decrease in negative surface charge density, as fewer ionizable oxygenated groups remain available for deprotonation, particularly under neutral to basic conditions. Conversely, rGO-AMN exhibits a pronounced increase in both oxygen content (O1s from 17.96k to 24.63k) and nitrogen-based functionalities (N1s from 6.5k to 49.1k), particularly graphitic and sp² nitrogen species.^[19][19,20]^ This compositional shift suggests a more functionally complex and amphoteric surface, where negatively charged carboxyl and quinone groups coexist with protonatable amine moieties. Such a configuration may enable greater colloidal stability and charge-switching behavior in aqueous environments, especially across varying pH conditions. Therefore, while CBX-functionalization favors a more hydrophobic and less ionizable surface post-sonication, AMN-functionalization introduces a chemically versatile interface with enhanced potential for biomolecular interaction and environmental responsiveness.

**Table S3**. Quantitative XPS deconvolution (D.A – deconvoluted area; D.S – deconvoluted signal. eV – binding energy) data for C 1s, O 1s, and N 1s regions of rGO-AMN and rGO-CBX before and after 1 h sonication

| **Sample** | **C1s %** | **D.S** | **eV** | **D.A** | **sp^2^ C / sp^3^ C** | **O1s %** | **D.S** | **eV** | **A** | **N1s %** | **D.S** | **eV** | **D.A** |
| --- | --- | --- | --- | --- | --- | --- | --- | --- | --- | --- | --- | --- | --- |
| rGO-AMN | 76.63 | sp^2^ C | 283.93 | 15293.60 | 2.29 | 15.02 | quinone | 529.27 | 258.71 | 8.35 | graphitic N | 398.59 | 560.26 |
|  |  | sp^3^ C | 284.65 | 6677.44 |  |  | C=O | 530.50 | 4603.49 |  | sp^2^ N | 399.94 | 4439.18 |
|  |  | C–OH / C–O–C | 285.36 | 7708.93 |  |  | O–C=O | 531.88 | 12717.59 |  | sp^3^ N | 401.57 | 1125.82 |
|  |  | C=O | 286.46 | 6712.05 |  |  | C–O–C | 533.38 | 2127.33 |  | H–N–H | 404.35 | 334.90 |
|  |  | O–C=O | 288.09 | 3544.83 |  |  | N–O / water | 535.06 | 1018.54 |  |  |  |  |
|  |  | π–π* | 290.68 | 1425.07 |  |  |  |  |  |  |  |  |  |
| rGO-AMN 1h | 75.93 | sp^2^ C | 283.75 | 19938.69 | 4.35 | 16.70 | quinone | 528.03 | 1536.91 | 6.72 | graphitic N | 399.32 | 4924.89 |
|  |  | sp^3^ C | 284.74 | 4586.32 |  |  | C=O | 528.74 | 5263.44 |  | sp^2^ N | 400.59 | 30273.69 |
|  |  | C–OH / C–O–C | 285.31 | 13175.18 |  |  | O–C=O | 530.32 | 14330.76 |  | sp^3^ N | 402.99 | 10053.05 |
|  |  | C=O | 286.66 | 5139.89 |  |  | C–O–C | 531.58 | 3806.84 |  | H–N–H | 406.79 | 3841.91 |
|  |  | O–C=O | 288.07 | 6311.18 |  |  | N–O / water | 534.01 | 1403.38 |  |  |  |  |
|  |  | π–π* | 291.29 | 2853.72 |  |  |  |  |  |  |  |  |  |
| rGO-CBX | 70.22 | sp^2^ C | 286.05 | 11912.40 | 3.12 | 28.09 | quinone | 532.46 | 12111.66 |  | | | |
|  |  | sp^3^ C | 286.33 | 3815.90 |  |  | C=O | 533.03 | 12782.20 |  |  |  |  |
|  |  | C–OH / C–O–C | 287.87 | 11049.39 |  |  | O–C=O | 533.69 | 6765.83 |  |  |  |  |
|  |  | C=O | 289.24 | 5472.89 |  |  | C–O–C | 534.97 | 4932.88 |  |  |  |  |
|  |  | O–C=O | 291.04 | 2045.85 |  |  | C–OH / water | 538.27 | 816.50 |  |  |  |  |
|  |  | π–π* | 293.55 | 515.98 |  |  |  |  |  |  |  |  |  |
| rGO-CBX 1 h | 70.65 | sp^2^ C | 286.53 | 28555.65 | 3.90 | 26.72 | quinone | 529.21 | 13549.31 |  |  |  |  |
|  |  | sp^3^ C | 287.46 | 7331.26 |  |  | C=O | 530.75 | 33369.75 |  |  |  |  |
|  |  | C–OH / C–O–C | 288.87 | 14738.28 |  |  | O–C=O | 532.05 | 7594.30 |  |  |  |  |
|  |  | C=O | 290.52 | 1504.69 |  |  | C–O–C | 534.19 | 3379.40 |  |  |  |  |
|  |  | O–C=O | 291.51 | 2303.23 |  |  | C–OH / water | 536.27 | 988.86 |  |  |  |  |
|  |  | π–π* | 293.75 | 462.14 |  |  |  |  |  |  |  |  |  |

TEM micrographs (Figure S6) illustrate the morphological evolution of rGO-CBX and rGO-AMN following 1 h of ultrasound-mediated dispersion, with clear distinctions in exfoliation degree, sheet conformation, and surface interactions. For rGO-CBX, large graphene domains are visible, consisting of well-defined lamellar structures with continuous, smooth surfaces and relatively uniform contrast. These compact carbonaceous deposits suggest a low degree of exfoliation and limited fragmentation. The absence of significant torsions or ruptures in these layers, coupled with their extended lateral dimensions, may indicate the preservation of turbostratically ordered multilayer assemblies. In this context, the term "turbostratic" refers to a quasi-crystalline stacking of graphene sheets with rotational misalignment and variable interlayer registry, a structural motif often retained in partially reduced GO species. Although individual sheets are discernible, they often remain interconnected, indicating a limited exfoliation efficiency and a tendency toward restacking, likely driven by interlayer π–π interactions in the absence of significant chemical disruption.


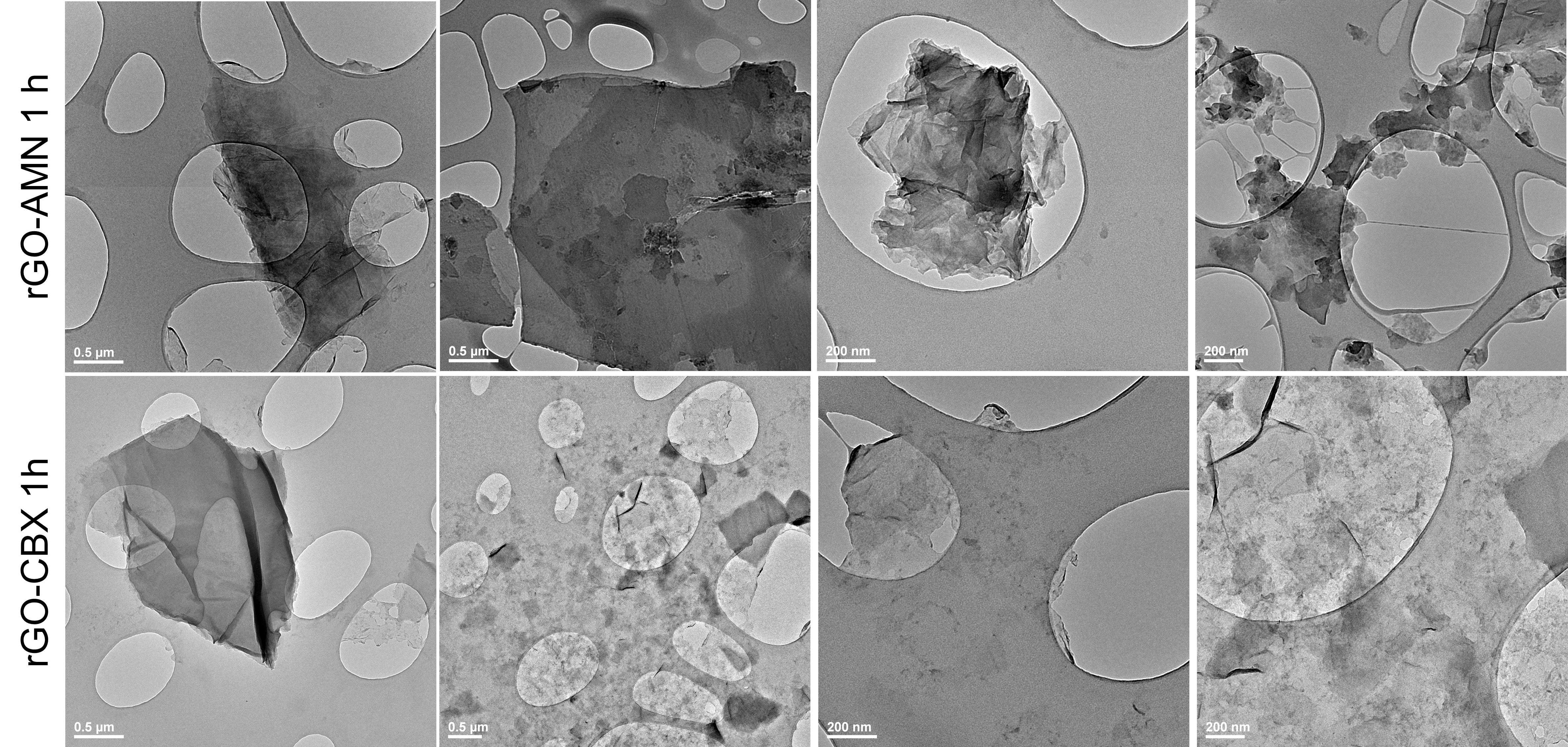


**Figure 6S.** TEM micrographs of rGO-CBX and rGO-AMN after 1 h of ultrasonication, illustrating distinct morphological features and exfoliation behavior. Scale bars represent 0.5 μm or 200 nm, as indicated.

On the other hand, the rGO-AMN sample subjected to the same 1 h sonication exhibits a more fragmented and dispersed morphology. Thin, wrinkled carbon structures dominate the field of view, many of which display folded edges and discontinuous outlines that may denote either nanoscale ruptures or monolayer formation. The presence of transparent zones with very faint contrast over the carbon grid strongly suggests the formation of ultrathin foils, potentially corresponding to few-layer or monolayer carbonaceous sheets. These appear to be arranged in irregular agglomerates or loosely packed, flexible morphologies, indicative of a more advanced exfoliation regime. Notably, the irregular folds, flexions, and distortions observed in these nanosheets align with the morphological criteria for turbostratic disorde characterized by rotational and translational misorientation between layers. This disorder contributes to a disrupted stacking periodicity, expected to manifest as broadened and downshifted (002) peaks in XRD patterns. The nanometric foils appear entangled yet poorly restacked, suggesting that AMN functionalization may enhance the colloidal stability and steric repulsion among graphene sheets, thereby maintaining their individual dispersion. Compared to CBX, AMN-based rGO displays a markedly more exfoliated state, with the emergence of thin-layered domains and a higher degree of topographic disorder.

TEM images provide direct insight into the lateral dimensions of rGO-AMN and rGO-CBX samples after 1 hour of sonication. The rGO-CBX sheets appear generally well-defined and planar, with lateral sizes predominantly ranging from approximately 400 nm to 1.2 µm, occasionally forming compact domains of even larger dimensions. In contrast, rGO-AMN exhibits a more fragmented and wrinkled morphology, with sheet sizes distributed between 300 and 800 nm, often forming overlapping structures and irregular assemblies. At higher magnification (scale bar 200 nm), nanometric features – particularly in the rGO-AMN sample – highlight extensive fragmentation, with individual flakes and discontinuities suggesting partial delamination or edge folding. These variations suggest that AMN functionalization may enhance sheet flexibility and hinder restacking, while CBX favors more continuous, multilayer structures. Both morphologies feature localized monolayer-like transparency, indicative of sonication-assisted exfoliation and partial disruption of van der Waals stacking.

The integrated structural, chemical, and morphological investigations indicate that 1 h sonicated rGO-AMN demonstrates enhanced exfoliation, elevated sp² character (and substantial nitrogen doping) relative to rGO-CBX. TEM imaging confirms the existence of thinner, more pliable sheets in AMN samples, indicating turbostratic stacking and potential monolayer formation, while XRD confirms the preservation of a higher degree of crystalinity. These attributes should provide better dispersion within biopolymer matrices owing to enhanced interfacial compatibility and diminished agglomeration. The enhanced surface functionality and increased π-conjugation in rGO-AMN likely promote superior cellular adhesion and interaction, particularly in *in vitro* static conditions, where, unlike *in vivo,* no host-mediated remodeling occurs, and the intrinsic micromechanical properties of the scaffold exclusively govern the biointerface dynamics. Nonetheless, *in vivo* results are influenced by a more intricate interaction of biological, mechanical, and immunological variables, which can modify or even override the effects seen in simplified *in vitro* environments.

**References**

1. Khramtsov P, Bochkova M, Timganova V, Nechaev A, Uzhviyuk S, Shardina K, et al. Interaction of graphene oxide modified with linear and branched PEG with monocytes isolated from human blood. Nanomaterials 2021;12(1):126.

2. Singh SB, Dastgheib SA. Characteristics of graphene oxide-like materials prepared from different deashed-devolatilized coal chars and comparison with graphite-based graphene oxide, with or without the ultrasonication treatment. Carbon N Y 2024;228:119331.

3. Andonovic B, Temkov M, Ademi A, Petrovski A, Grozdanov A, Paunović P, et al. Laue functions model vs Scherrer equation in determination of graphene layers number on the ground of XRD data. Journal of Chemical Technology and Metallurgy 2014;49(6):545–50.

4. Tuz Johra F, Lee J, Jung WG. Facile and safe graphene preparation on solution based platform. Journal of Industrial and Engineering Chemistry 2014;20:2883–2887.

5. Tyurnina A V, Morton JA, Subroto T, Khavari M, Maciejewska B, Mi J, et al. Environment friendly dual-frequency ultrasonic exfoliation of few-layer graphene. Carbon N Y 2021;185:536–45.

6. Emiru TF, Ayele DW. Controlled synthesis, characterization and reduction of graphene oxide: A convenient method for large scale production. Egyptian Journal of Basic and Applied Sciences 2017;4(1):74–9.

7. Chua CK, Pumera M. Regeneration of a conjugated sp2 graphene system through selective defunctionalization of epoxides by using a proven synthetic chemistry mechanism. Chemistry–A European Journal 2014;20(7):1871–7.

8. Jokar S, Pourjavadi A, Adeli M. Albumin-graphene oxide conjugates; Carriers for anticancer drugs. RSC Adv 2014;4.

9. Khalili D. Graphene oxide: a promising carbocatalyst for the regioselective thiocyanation of aromatic amines, phenols, anisols and enolizable ketones by hydrogen peroxide/KSCN in water. New Journal of Chemistry 2016;40(3):2547–53.

10. Tuz Johra F, Lee J, Jung WG. Facile and safe graphene preparation on solution based platform. Journal of Industrial and Engineering Chemistry 2014;20:2883–2887.

11. Kokmat P, Surinlert P, Ruammaitree A. Growth of high-purity and high-quality turbostratic graphene with different interlayer spacings. ACS Omega 2023;8(4):4010–8.

12. Girgis BS, Temerk YM, Gadelrab MM, Abdullah ID. X-ray diffraction patterns of activated carbons prepared under various conditions. Carbon science 2007;8(2):95–100.

13. Pavoski G, Maraschin T, Fim F de C, Balzaretti NM, Galland GB, Moura CS, et al. Few layer reduced graphene oxide: evaluation of the best experimental conditions for easy production. Materials Research 2016;20:53–61.

14. Solati N, Mobassem S, Kahraman A, Ogasawara H, Kaya S. A comprehensive study on the characteristic spectroscopic features of nitrogen doped graphene. Appl Surf Sci 2019;495:143518.

15. Novoa-De Leon IC, Johny J, Vazquez-Rodriguez S, Avellaneda-Avellaneda D, Shaji S, Sepúlveda-Guzmán S. Nanocarbon Hybrid Films of Reduced Graphene Oxide and N-Doped Graphene Quantum Dots as a Metal-Free Platform for Graphene-Enhanced Raman Scattering. ACS Appl Mater Interfaces 2025;17(11):17251–9.

16. Wang S, Dong Y, He C, Gao Y, Jia N, Chen Z, et al. The role of sp 2/sp 3 hybrid carbon regulation in the nonlinear optical properties of graphene oxide materials. RSC Adv 2017;7(84):53643–52.

17. Novoa-De Leon IC, Johny J, Vazquez-Rodriguez S, Avellaneda-Avellaneda D, Shaji S, Sepúlveda-Guzmán S. Nanocarbon Hybrid Films of Reduced Graphene Oxide and N-Doped Graphene Quantum Dots as a Metal-Free Platform for Graphene-Enhanced Raman Scattering. ACS Appl Mater Interfaces 2025;17(11):17251–9.

18. Fauzi F, Azizi F, Musawwa MM, Dwandaru WSB. Synthesis and characterisations of reduced graphene oxide prepared by microwave irradiation with sonication. Journal of Physical Science 2021;32(2):1–13.

19. Kovtun A, Jones D, Dell’Elce S, Treossi E, Liscio A, Palermo V. Accurate chemical analysis of oxygenated graphene-based materials using X-ray photoelectron spectroscopy. Carbon N Y 2019;143:268–75.

20. Liu W, Speranza G. Tuning the oxygen content of reduced graphene oxide and effects on its properties. ACS Omega 2021;6(9):6195–205.
